# SDCBP Modulates Stemness and Chemoresistance in Head and Neck Squamous Cell Carcinoma through Src Activation

**DOI:** 10.3390/cancers13194952

**Published:** 2021-10-01

**Authors:** Cristina Mir, Yoelsis Garcia-Mayea, Laia Garcia, Pol Herrero, Nuria Canela, Rocío Tabernero, Juan Lorente, Josep Castellvi, Eva Allonca, Juana García-Pedrero, Juan Pablo Rodrigo, Ángel Carracedo, Matilde Esther LLeonart

**Affiliations:** 1 Biomedical Research in Cancer Stem Cells Group, Vall d’Hebron Research Institute (VHIR), 08035 Barcelona, Spain; cristina.mir@vhir.org (C.M.); yoelsis.garcia@vhir.org (Y.G.-M.); laia.garcia@hotmail.com (L.G.); joscastellvi@vhebron.net (J.C.); 2 Faculty of Medicine, University of Barcelona, 08036 Barcelona, Spain; 3 Eurecat, Centre Tecnològic de Catalunya–Centre for Omic Sciences (COS), Joint Unit Universitat Rovira i Virgili-EURECAT, 43204 Reus, Spain; pol.herrero@eurecat.org (P.H.); nuria.canela@eurecat.org (N.C.); 4 Otorhinolaryngology Department, Hospital Vall d’Hebron (HUVH), Universitat Autònoma de Barcelona (UAB), 08035 Barcelona, Spain; rtaberne@vhebron.net (R.T.); jlorente@vhebron.net (J.L.); 5 Department of Otolaryngology, Hospital Universitario Central de Asturias (HUCA), Instituto de Investigación Sanitaria del Principado de Asturias, IUOPA, University of Oviedo, 33011 Oviedo, Spain or ynkc1@hotmail.com (E.A.); juanagp@ispasturias.es (J.G.-P.); jprodrigo@uniovi.es (J.P.R.); 6 Spanish Biomedical Research Network Centre in Oncology, CIBERONC, Av. Monforte de Lemos, 3-5. Pabellón 11. Planta 0, 28029 Madrid, Spain; 7 Fundación Pública Galega de Medicina Xenómica, Instituto de Investigación Sanitaria de Santiago de Compostela (IDIS), Complejo Hospitalario Universitario de Santiago, SERGAS, 15706 Santiago de Compostela, Spain; angel.carracedo@usc.es

**Keywords:** HNSCC, chemoresistance, cancer stem cells, stemness, SDCBP

## Abstract

**Simple Summary:**

Drug resistance is the principal limiting factor to achieving good survival rates in patients with cancer. The identification of potential biomarkers for diagnosis and prognostic prediction, as well as the design of new molecular-targeted treatments, will be essential to improving head and neck squamous cell carcinoma (HNSCC) patient outcomes. In this sense, the sensitization of resistant cells and cancer stem cells (CSCs) represents a major challenge in cancer therapy. We conducted a proteomic study involving cisplatin-resistance and CSCs with the aim to unravel the molecular and cellular mechanisms by which tumor cells acquire resistance to chemotherapy. Syntenin-1 (SDCBP) was identified as an important protein involved in the chemoresistance and stemness of HNSCC tumors.

**Abstract:**

To characterize the mechanisms that govern chemoresistance, we performed a comparative proteomic study analyzing head and neck squamous cell carcinoma (HNSCC) cells: CCL-138 (parental), CCL-138-R (cisplatin-resistant), and cancer stem cells (CSCs). Syntenin-1 (SDCBP) was upregulated in CCL-138-R cells and CSCs over parental cells. SDCBP depletion sensitized biopsy-derived and established HNSCC cell lines to cisplatin (CDDP) and reduced CSC markers, Src activation being the main SDCBP downstream target. In mice, SDCBP-depleted cells formed tumors with decreased mitosis, Ki-67 positivity, and metastasis over controls. Moreover, the fusocellular pattern of CCL-138-R cell-derived tumors reverted to a more epithelial morphology upon SDCBP silencing. Importantly, SDCBP expression was associated with Src activation, poor differentiated tumor grade, advanced tumor stage, and shorter survival rates in a series of 382 HNSCC patients. Our results reveal that SDCBP might be a promising therapeutic target for effectively eliminating CSCs and CDDP resistance.

## 1. Introduction

Head and neck squamous cell carcinoma (HNSCC) is the sixth most common cancer in the world and can affect the oral cavity, pharynx, larynx, paranasal sinuses, nasal cavity, or salivary glands [1,2]. HNSCC is generally curable when diagnosed early; however, patients are often diagnosed at an advanced and less curable stage, requiring more aggressive treatment and resulting in severe consequences and reduced life expectancy. In fact, the 5-year survival rates for HNSCC patients have remained at ~50% over the last 40 years, largely due to frequent tumor recurrence and/or metastatic dissemination [3,4]. Therefore, the identification of potential biomarkers for diagnosis and prognostic prediction, as well as the design of new molecular-targeted treatments, will be essential to improving HNSCC patient outcomes.

The rapid evolution of HNSCC suggests the acquisition of resistance phenotypes by cancer cells, as well as the emergence of novel genetic/molecular alterations, in a dynamic, permanently active, and mutagenic tumor microenvironment [5]. The existence of cancer stem cells (CSCs) responsible for tumor initiation and progression, resistance to therapies, metastasis, and relapses have been described in many tumors [2,6,7,8]. CSC properties include the ability to self-renew, formation of tumorspheres in nonadherent culture conditions, expression of specific cell surface receptors (such as CD44 and CD133 in HNSCC), expression of transcription factors related to pluripotency (Nanog, Sox-2, and Oct-3/4), and high expression of ABC transporters.

A comparative proteomic analysis revealed syntenin-1, also known as syndecan-binding protein (SDCBP) or melanoma differentiation-associated gene-9 (MDA-9), to be one of the proteins most highly overexpressed in CCL-138-R cells and CSCs compared to CCL-138 cells. Since its discovery, SDCBP was implicated in melanoma metastasis and associated with multiple cellular functions, including cell-cell interactions, cell-matrix adhesion, signal transduction, and cell trafficking [9,10]. Its role in proliferation, migration, invasion, differentiation, and angiogenesis in HNSCC is currently described [11,12]. In the present study, we focused on thoroughly investigating the clinical and biological relevance of SDCBP in the stemness and chemoresistance properties in HNSCC. The impact of SDCBP inhibition in in vitro and in vivo HNSCC models, together with its clinical and prognostic relevance in patient biopsies, strongly supports a central role of SDCBP in HNSCC.

## 2. Materials and Methods

### 2.1. Cell and Tissue Culture

Primary tumors from patients with larynx squamous cell carcinoma were collected from Vall d’Hebron University Hospital (HUVH) for cell culture and protein analysis. The establishment of a primary cell culture from these laryngeal biopsies was performed as previously described [13,14]. The study was performed according to the Declaration of Helsinki and approved by the Ethics Committee of Vall d’Hebron Hospital (CEIC) (Ref. PR(AG)342/2016). None of the patients enrolled in this study received any radio- or chemotherapeutic treatment prior to surgical intervention. Informed consent was obtained from all the patients.

HNSCC cell lines derived from pharyngeal (HTB-43 [FaDu] and CCL-138 [Detroit 562]), laryngeal (JHU029 [CVCL-5993]), and tongue (SCC-25 [CRL-1628]) carcinomas were studied. The HTB-43 and CCL-138 cell lines were obtained from the American Type Culture Collection (ATCC, Manassas, VA, USA), and the JHU029 and SCC-25 cells were kindly provided by Dr. Aznar-Benitah (IRB). These cell lines were all authenticated based on polymorphic short tandem repeat loci. All the cell lines were cultured in MEM (Gibco, Thermo Fisher Scientific, Waltham, MA, USA) supplemented with 10% fetal bovine serum (FBS) (Biowest, Nuaillé-France), 1% penicillin-streptomycin (Pen 20 U/mL and Strep 20 μg/mL, Gibco, Thermo Fisher, Waltham, MA, USA). Resistant cell lines and CSCs were generated as described [14].

### 2.2. Proteomic Analysis

Protein extracts from parental, resistant, and CSC subpopulations derived from CCL-138 cells were analyzed at the Centre for Omic Sciences (COS-Eurecat, Tarragona, Spain) as previously described [14,15,16]. Briefly, protein extracts were analyzed in a comparative way by gel free proteomics technology (TMT-nLC-Orbitrap) using isobaric labeling by Tandem Mass Tag (TMT) of the peptides and multidimensional fractionation, first by the isoelectric point (off Gel) and then by reverse phase nanochromatography coupled with high resolution mass spectrometry (nanoLC-Orbitrap). The mass spectrometry proteomics data were deposited to the ProteomeXchange Consortium via the PRIDE partner repository, dataset identifier PXD020159.

### 2.3. Transfection of Small Interfering RNAs (siRNAs)

Cells were counted and prepared in a medium without antibiotics at a density of 300,000 cells/well in 6-well plates. In parallel, siRNAs targeting SDCBP (ID:3686, siSDCBP, siTOOLs Biotech, Planegg, Germany), Src (ID:6714, siSrc, siTOOLs Biotech, Planegg, Germany), or the control, siPOOL (siCtrl) (siTOOLs Biotech, Planegg, Germany), were used for transfection (3 nM/well) with Lipofectamine RNAiMAX (Invitrogen, Thermo Fisher Scientific, Waltham, MA, USA). An additional siRNA for SDCBP (ID:hs.Ri.SDCBP.13.1, 2siSDCBP, Integrated DNA Technologies, Coralville, IA, USA) or the control (ID:DS NC1; 2siCtrl, Integrated DNA Technologies, Coralville, IA, USA) (Integrated DNA Technologies, Coralville, IA, USA) was used for transfection (5 nM/well).

### 2.4. SDCBP Cloning and Overexpression

RNA was extracted from CCL-138-R cells, followed by qRT-PCR (SuperScript III One-Step RT-PCR System with Platinum TaqHigh Fidelity DNA Polymerase, Invitrogen, Thermo Fisher Scientific, Waltham, MA, USA) and amplification (Q5 Hot-Start DNA Polymerase, New England Biolabs, Werfen, Barcelona, Spain). After 2 h of digestion of the pIRES2-EGFP vector with Nhe I and Sal I (New England Biolabs), alkaline phosphatase treatment (FastAP Thermosensitive Alkaline Phosphatase, Thermo Fisher Scientific, Waltham, MA, USA), overnight ligation at 16 °C (T4 DNA ligase, New England Biolabs, Ipswich, MA, USA), and the transformation of DH5α cells, the DNA plasmid was obtained with the PureLink HiPure Plasmid MidiPrep Kit (#K210005, Invitrogen, Thermo Fisher Scientific, Waltham, MA, USA).

Cells were seeded at a density of 600,000 cells/well in 6-well plates. The next day, in parallel, the cells were transduced with pIRES2-EGFP (indicated as pIRES2-EGFP or pIRES2-EGFP-SDCBP when the ORF sequence of SDCBP was inserted (3 µg/well)) or pcDNA3.1+/C-(K)DYK (GenScript Biotech, Leiden, The Netherlands) (indicated as pcDNA or pcDNA-SDCBP when the ORF sequence of SDCBP was inserted (4 μg/well)) and Lipofectamine 3000 (Invitrogen, Thermo Fisher Scientific, Waltham, MA, USA). In the case of pIRES2-EGFP transfection, antibiotic selection was performed over 7 days with 2 mg/mL of G418.

### 2.5. Western Blot

Normal and tumor tissue samples, as well as HNSCC cell lines, were lysed with the RIPA buffer (25 mM TrisCl, 150 mM NaCl, 1% Igepal, 1% sodium deoxycholate, 0.1% SDS, pH 7.5) and a phosphatase inhibitor cocktail (Thermo Fisher Scientific, Waltham, MA, USA). Protein lysates (50 μg) were loaded in precast polyacrylamide gels (Bolt 4–12% Bis-Tris Plus Gels, Invitrogen, Thermo Fisher Scientific, Waltham, MA, USA). The membranes were incubated overnight at 4 °C with the following antibodies: SDCBP (#ab133267, Abcam, Cambridge, UK), p-Src (Tyr416) (#6943, Cell Signaling Technology Europe Leiden, The Netherlands), Src (#2109, Cell Signaling Technology, Europe Leiden, The Netherlands), CD44 (GTX102111, GeneTex, Labclinics, Barcelona, Spain), Sox2 (GTX101507, GeneTex, Barcelona, Spain), Vinculin (sc-73614, Santa Cruz Biotechnology, Heidelberg, Germany), and β-actin (#A3854, Sigma-Aldrich, Merck, Madrid, Spain). Then, the bands were visualized as described [14].

### 2.6. Drug Assay

Cells were seeded in 96-well plates at a density of 20,000 cells/well. The next day, the cells were exposed in triplicate to different concentrations of CDDP (Sigma-Aldrich, Merck, Madrid, Spain) (0–150 μM) or dasatinib (Sigma-Aldrich, Merck, Madrid, Spain) (5–100 nM) for 72 h. To determine the number of viable cells, the cell metabolic activity was assessed using MTS (CellTiter 96 AQueous MTS Reagent Powder, Promega Biotech Iberica, Madrid, Spain), as indicated by the manufacturers. Survival curves and IC50 calculations were performed using GraphPad Prism (GraphPad Software, San Diego, CA, USA). A representative experiment of at least 3 independently performed experiments is shown.

### 2.7. Tumorsphere Formation

Transfected cells were seeded at a density of 10,000 cells/mL in nonadherent conditions using 48-well plates coated with poly-HEMA (Sigma-Aldrich, Merck, Madrid, Spain). Tumorsphere growth in the stem cell selection medium (3D Tumorsphere medium XF, PromoCell, Sigma-Aldrich, Merck, Madrid, Spain) was monitored for 10 days, and the stem cell medium was supplied every 3–4 days. At the endpoint, spheres from 3 independent experiments were counted manually in triplicate. The tumorsphere area was measured from pictures (10×) with ImageJ software, and the means are represented in graphs.

### 2.8. Quantitative Real-Time PCR (qRT-PCR)

RNA extraction was performed using the mirVana miRNA Isolation Kit (Ambion, Austin, TX, USA), and RNA was treated with DNAse I using the DNA-free DNA Removal Kit (Invitrogen, Thermo Fisher Scientific, Waltham, MA, USA), according to the manufacturer’s instructions. RNA quality was assessed by analyzing RNA integrity number (RIN) values with an Agilent 2100 Bioanalyzer system. cDNA was obtained using the RevertAid H Minus First Strand cDNA Synthesis Kit (Thermo Fisher Scientific, Waltham, MA, USA). mRNA expression was analyzed using TaqMan probes (Thermo Fisher Scientific, Waltham, MA, USA) for the following genes: Nanog (Hs04399610_g1), Sox2 (Hs01053049_s1), Oct3/4 (Hs04260367_gH), CD133/PROM1 (Hs01009257_m1), CD44 (Hs01075861_m1), ABCB1 (Hs00184500_m1), KLF4 (Hs00358836_m1), N-cadherin (Hs00983056_m1), E-cadherin (Hs01023894_m1), Slug (Hs00161904_m1), Twist (Hs01675818_s1), and the endogenous genes TBP (Hs00427620_m1) and IPO8 (Hs00183533).

### 2.9. Flow Cytometry

A side population (SP) study was performed as previously described with slight modifications [17]. Briefly, 48 h after transfection, cells were washed with PBS and resuspended at a density of 1 × 10^6^ cells/mL in a medium supplemented with 2% FBS and 10 mM 4-(2-hydroxyethyl)-1-piperazineethanesulfonic acid (HEPES) (Sigma-Aldrich, Merck, Madrid, Spain). Then, the cells were incubated at 37 °C for 120 min with 5 μg/mL Hoechst 33342 dye (Thermo Fisher Scientific, Waltham, MA, USA). The control cells were incubated at 37 °C with 50 μM verapamil (Sigma-Aldrich, Merck, Madrid, Spain) for 15 min before the addition of Hoechst dye to validate SP detection. For dead cell discrimination, propidium iodide (PI) (Sigma-Aldrich, Merck, Madrid, Spain) at 5 μg/mL was added to the cells before FACS analysis on a FACSAria II flow cytometer (BD Biosciences, San Jose, CA, USA).

Regarding ABCB1 detection, 1 × 10^6^ cells/mL were washed, resuspended in PBS, and incubated at 4 °C for 20 min in PBS with 2% FBS. Further, the cells were incubated at room temperature (RT) (darkness) for 30 min with the ABCB1/Mdr1-PE antibody (1 μg/mL) (#sc55510, 200 μg/mL, Santa Cruz Biotechnology, Heidelberg, Germany). PI was added at 5 μg/mL before FACS analysis on the LSR Fortessa flow cytometer (BD Biosciences).

Apoptosis detection was performed with the Annexin V-APC Detection Kit (Thermo Fisher Scientific, Waltham, MA, USA) according to the manufacturer’s instructions. Briefly, 1 × 10^6^ cells/mL were washed in cold PBS and resuspended in 100 μL of 1× binding buffer. Further, they were incubated for 15 min at RT with 5 μL of Annexin V-APC, washed, and resuspended in 400 μL of 1× binding buffer. Lastly, PI was added at 5 μg/mL, and cells were then analyzed on the LSRFortessa (BD Biosciences, CA, USA).

The results were confirmed in 3 independent experiments.

### 2.10. Animal Studies

All the experimental protocols were performed in accordance with the institutional Animal Research Ethical Committee (CEEA Ref. 42/15). In all cases, 6- to 7-week-old female athymic NMRI-Foxn1nu/nu mice (Janvier LabsSaint Berthevin Cedex, Le Genest-Saint-Isle, France) were subcutaneously inoculated in their flanks. For the CDDP resistance acquisition model, the mice were injected with HTB-43 or CCL-138 cells (1 × 10^5^ cells/injection) mixed 1:1 with Matrigel (Corning, Thermo Fisher Scientific, Waltham, MA, USA). Once the tumors reached 1.5 mm^3^, the mice that received each cell line were randomized into two groups (N = 10 mice/group): (i) vehicle (DMSO); and (ii) 2 or 3 intraperitoneal injections of CDDP (1.5 mg/mL and 6 mg/mL) according to modifications of a protocol described elsewhere and adapted for each cell line [18]. The tumor growth was measured using digital calipers (Mitutoyo, Thermo Fisher Scientific, Waltham, MA, USA) and the formula (length (mm) × width^2^ (mm) × π)/6, 3 times/week (>20% of weight loss was considered the endpoint criterion). Primary cell cultures were established from the collected tumors. To analyze tumor growth rates in vivo, parental cells, CDDP-resistant cells (generated from one resistant tumor (confirmed by drug assay)), and CSCs from the HTB-43 and CCL-138 cell lines (1 × 10^5^ cells/injection) were inoculated into mice (4 tumors/group). The tumors and organs were collected for Hematoxylin and eosin, Ki-67 (NCL-L-Ki67-MM1, Leica Biosystems, Barcelona, Spain), and p63 staining (HPA006288, Atlas Antibodies, Quimigen, Madrid, Spain).

In contrast, 1 × 10^6^ transfected JHU029 and JHU029-R cells (siCtrl and siSDCBP conditions) were inoculated into mice in the following groups (8 tumors/group): (i) JHU029 cells siCtrl; (ii) JHU029 cells siSDCBP; (iii) JHU029-R cells siCtrl; and (iv) JHU029-R cells siSDCBP. The mice were monitored by fluorescence preclinical imaging using an IVIS Spectrum In Vivo Imaging System (Perkin Elmer, Waltham, MA, USA) for the detection of metastasis as previously described [14].

### 2.11. Patients

Surgical tissue specimens from 382 patients diagnosed with HNSCC at the Hospital Universitario Central de Asturias (HUCA) were retrospectively collected in accordance with the Institutional Ethics Committee of the HUCA (Ref. 141/19). Informed consent was obtained from all the patients. The patients were surgically treated for a single primary tumor and received no treatment prior to surgery. No patients had distant metastasis at the time of diagnosis. Tumors were staged according to the TNM system [19].

Three 1-mm cylinders were obtained from each paraffin-embedded tumor to construct TMA blocks, including a total of 249 oropharyngeal, 65 hypopharyngeal, and 68 laryngeal SCCs. Each TMA included 3 cores of normal epithelium (pharyngeal and laryngeal mucosa obtained from nononcologic patients). Only 10 (3%) HPV-positive tumors (8 oropharyngeal, 1 laryngeal, and 1 hypopharyngeal) were detected using p16 immunohistochemistry, high-risk HPV DNA detection by in situ hybridization, and genotyping by GP5+/6+-PCR, as previously reported [20].

### 2.12. Immunohistochemistry (IHC)

IHC staining was performed at RT on an automatic staining workstation (Dako Autostainer Plus, Glostrup, Denmark) using anti-SDCBP (ab133267, Abcam, Cambridge, UK), anti-p21 (Clone 4D10; Leica Biosystems NCL-L-WAF-1, Barcelona, Spain) at a 1:10 dilution, anti-E-cadherin (BD Biosciences, CA, USA) at a 1:4000 dilution, anti-Src (active) Clone 28 (#AHO0051, Thermo Fisher Scientific, Waltham, MA, USA) at a 1:300 dilution, anti-Ki67 (clone MIB-1, Dako, Glostrup, Denmark) for 30 min, and anti-Nanog and anti-Notch antibodies, as described. [21] Counterstaining with Hematoxylin and eosin was the final step. For the Ki-67, p21, and Notch1 proteins, nuclear staining was evaluated and dichotomized as a negative expression (0–10% stained cells) versus positive expression (>10% stained tumor cells). E-cadherin, Src, Nanog, and Notch1 were scored as described [14,21]. Validation of SDCBP was performed at the nuclear, membrane, and cytoplasmic levels in each patient according to the immunoreactive score (IRS) by multiplying the quantity and staining intensity scores (Table S1).

### 2.13. Statistical Analysis

For the proteomic study, a one-way ANOVA for all cell groups was performed, followed by the Student’s *t*-test for CCL-138 CSCs versus CCL-138 cells and CCL-138-R cells versus CCL-138 cells. For qRT-PCR, transfection, and mouse experiments, the Student’s *t*-test was performed to compare the differences between two groups. For IHC, to determine the correlation between the expression of SCDBP, active Src, E-cadherin, Ki-67, p21, Notch1, and Nanog, the Mann–Whitney U test, Pearson’s correlation, or the Kruskal–Wallis test was performed according to continuous or dichotomous variables. Kaplan–Meier curves were generated to determine disease-free survival and overall survival in relation to SCDBP expression (SPSS 15.0 software package, SPSS Inc., Chicago, IL, USA).

## 3. Results

### 3.1. SDCBP Expression in HNSCC Cell Lines

**Figure 1:**
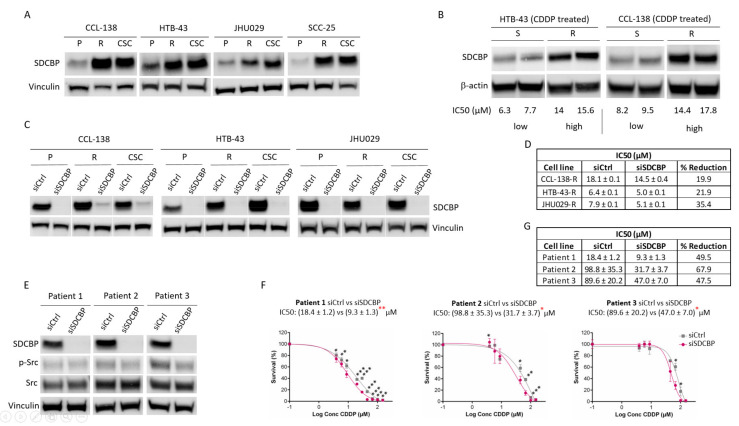
SDCBP is involved in resistance. (**A**) Western blot of SDCBP expression in HNSCC cell lines: parental cells (P), CDDP-resistant cells (R), and cancer stem cells (CSCs) derived from each corresponding parental cell line; (**B**) Western blot of SDCBP expression in established cell lines derived from tumors of mice treated for one month with CDDP. At the experimental endpoint, the tumors were classified as sensitive (S) or resistant (R) tumors based on the IC50 values of CDDP (more information in Figure S2A–D); (**C**) Western blot of SDCBP levels in parental cells (P), CDDP-resistant cells (R), and cancer stem cells (CSCs) derived from HNSCC cell lines under conditions of SDCBP depletion; (**D**) Table of IC50 values of siSDCBP CDDP-resistant cells (R) from the indicated HNSCC cell lines; (**E**) Western blot of SDCBP expression and (**F**) survival curves of the IC50 values in patient-derived HNSCC cell lines, in which SDCBP was depleted. (**G**) Table indicating the IC50 values of Figure 1F. The reduction in the IC50 is indicated and describes the percentage of IC50 reduction in SDCBP-depleted cells versus control cells. * *p* < 0.05, ** *p* < 0.01, *** *p* < 0.001.

To gain insight into the mechanisms that govern HNSCC chemoresistance, we used a proteomic approach to compare the proteins differentially expressed in CCL-138 parental cells versus CCL-138-R cells and CSCs (the lists of all proteins deregulated, as well as the results of the statistical analyses performed, can be found in Tables S2–S5 of our previous report) [14]. A total of 36 dysregulated proteins were commonly observed in CCL-138-R cells and CSCs versus CCL-138 cells. Among these proteins, 12 were upregulated, and 24 were downregulated (Figure S1) [14]. One of the most highly upregulated proteins was SDCBP. To validate the proteomic results, SDCBP expression in CCL-138, HTB-43, JHU-029, and SCC-25 cells was first analyzed by Western blot. SDCBP upregulation in CDDP-resistant cells and in CSCs from each cell line compared to parental cells was confirmed (Figure 1A). To investigate whether SDCBP was involved in the acquisition of CDDP resistance in vivo, we generated a spontaneous model of CDDP resistance with tumors formed by CCL-138 and HTB-43 cells treated with CDDP (Figure S2A,B). The mice were sacrificed, and the tumor explants were grown in a culture to determine whether the tumors were sensitive or resistant according to the CDDP IC50 values (Figure S2C,D). The role of SDCBP in chemoresistance was confirmed in vivo by SDCBP expression in sensitive (low IC50) and resistant (high IC50) tumors derived from HTB-43 and CCL-138 cells. The tumors that showed higher IC50 values also exhibited higher SDCBP levels than tumors with low IC50 values (Figure 1B). All results were confirmed in three independent experiments. Moreover, we determined the ability of CCL-138 cells, CCL-138-R cells, HTB-43 cells, HTB-43-R cells, and CSCs to form tumors in vivo. Tumors formed by HTB-43-R cells, CCL-138-R cells, and CSCs grew more slowly than those formed by HTB-43 and CCL-138 cells (Figure S3A). We also found that tumors formed by CCL-138-R cells and CSCs had a more pronounced fusocellular phenotype than tumors formed by parental cells (Figure S3B), but no differences were seen in tumors formed by HTB-43 cells. Moreover, compared with mice injected with resistant or parental cells, mice injected with HTB-43 CSCs showed metastasis in the lung (Figure S3C). Therefore, the tumors formed by resistant cells and CSCs showed high SDCBP levels and were slow growing but more aggressive, which are common features in the clinical characteristics of drug resistance [22].

### 3.2. SDCBP Inhibition Sensitizes HNSCC Cells to CDDP

To determine the effect of SDCBP on the response to CDDP, SDCBP expression was inhibited by siRNA transfection into CCL-138, HTB-43, JHU029, and SCC-25 cells (Figure 1C and Figure S4A). Moreover, resistant cell lines showed that SDCBP inhibition sensitized cells to CDDP (Figure 1D and Figure S4B–D). The sensitization levels in SDCBP-inhibited cells varied between 10% and 36% compared with those in control cells, with SDCBP inhibition having a major sensitization effect in JHU029-R cells. This effect of SDCBP on the CDDP response was further confirmed using a different siRNA against SDCBP (Figure S5A–C). In this case, the IC50 was reduced by 19.4%, similar to that observed in HTB-43 cells with the previous siSDCBP (22%, Figure 1D). In addition, we selected three HNSCC-biopsy-derived cell lines with high CDDP resistance (>10 µM) as described [23] to test the effect of SDCBP inhibition on the CDDP response (Figure 1E). The IC50 values for CDDP were decreased by 31.6–58% compared with those observed in the control cells (Figure 1F,G). SDCBP inhibition showed a greater sensitizing effect on biopsy-derived HNSCC cell lines than on established HNSCC cells.

### 3.3. SDCBP Depletion Affects CSCs

**Figure 2:**
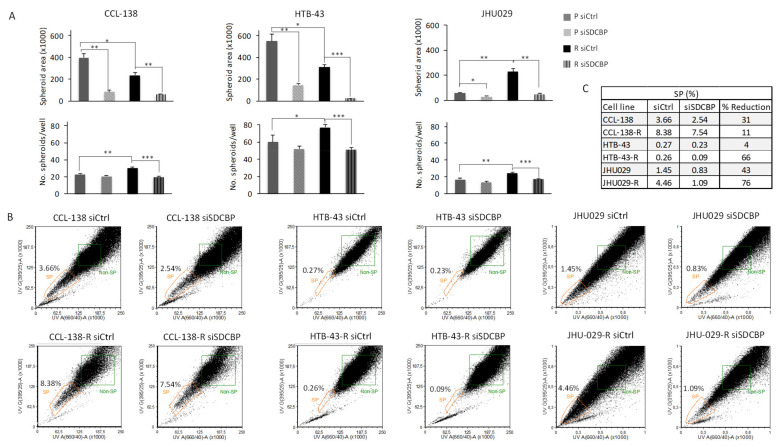
Analysis of CSC population in HNSCC cell lines. (**A**) The sphere formation abilities of parental (P) and resistant (R) cells transfected with siSDCBP were analyzed in 3D cell culture (nonadherent conditions and selective 3D tumorsphere medium). The plots represent the area of the spheres and number of spheres generated under the indicated conditions; (**B**) Flow cytometry plots of side population (SP) detection in the indicated cell lines under SDCBP depletion; (**C**) Percentages of CSCs given by the SP fraction detected in Figure 2B are shown with the percentages of reduction. One experiment is shown from at least 3 independent experiments. * *p* < 0.05, ** *p* < 0.01, *** *p* < 0.001.

We assessed the potential impact of SDCBP depletion on tumorsphere formation by CCL-138, CCL-138-R, HTB-43, HTB-43-R, JHU029, and JHU029-R cells. SDCBP expression was stably knocked down up to 9–15 days post-transfection (Figure S6A). The tumorsphere area analysis of SDCBP-depleted cells showed that the tumors were smaller than those formed by the corresponding control cells (Figure 2A upper panel, Figure S6B). Moreover, the number of tumorspheres formed by CDDP-resistant cells increased compared to those formed by parental cells for all the HNSCC cell lines. SDCBP depletion also decreased the number of tumorspheres formed by CCL-138-R, HTB-43-R, and JHU029-R cells (Figure 2A lower panel). These observations were confirmed in CCL-138-R and HTB-43-R cells using an independent siRNA against SDCBP (Figure S5D).

CSCs in HNSCC cells were measured by a side population (SP) analysis [24]. Upon SDCBP inhibition, an important decrease in the SP percentage was observed in CCL-138, CCL-138-R, HTB-43-R, JHU029, and JHU029-R cells (Figure 2B,C). No differences were observed in the SP of SCC-25 cells upon SDCBP depletion (data not shown). The CDDP sensitization observed in resistant cells upon SDCBP depletion seems to be associated with a reduction in the number of CSCs. Consequently, these results demonstrate that SDCBP inhibition reduces tumorsphere formation capacity and CSC subpopulations, suggesting that SDCBP could be involved in the regulation of CSC properties.

### 3.4. SDCBP Inhibition Decreases CSC-Related Genes

**Figure 3:**
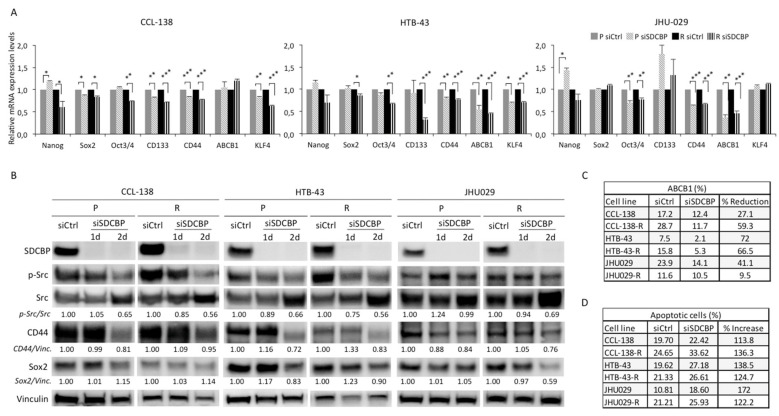
SDCBP depletion is associated with stemness. (**A**) HNSCC cell lines (parental, P, and CDDP-resistant, R, cells) were depleted of SDCBP and analyzed by qRT-PCR to determine the mRNA expression of the indicated stem cell genes. The most significant differences were found in CD44 for all the cell lines, followed by ABCB1 in HTB-43 and JHU029 cells (parental and resistant cells) and Notch1 in all the cell lines except the HTB-43 cells; (**B**) Western blot of cells analyzed in Figure 3A. Quantification of SDCBP/Vinculin, p-Src/Src ratio, CD44/Vinculin, and Sox2/Vinculin expression is indicated. Note that the p-Src/Src ratio decreases when SDCBP is inhibited, followed by CD44 and Sox2; (**C**) Analysis of ABCB1 expression by flow cytometry (plots are shown in Figure S5A); (**D**) The indicated HNSCC cell lines were depleted of SDCBP, and apoptosis induction was analyzed. The table shows the percentage of apoptosis, including both early apoptosis and late apoptosis (corresponding plots are shown in Figure S6). * *p* < 0.05, ** *p* < 0.01, *** *p* < 0.001.

We studied the effect of SDCBP depletion on the mRNA expression of genes known to regulate stemness: Nanog, Sox2, Oct3/4, CD133, CD44, ABCB1, and KLF4. CD44 expression decreased in all the cell lines upon SDCBP depletion (Figure 3A). Similarly, CD133 levels decreased upon SDCBP depletion in CCL-138, CCL-138-R, and HTB-43-R cells, while ABCB1 mRNA decreased in SDCBP-depleted HTB-43, HTB-43-R, and JHU029 cells, and KLF4 mRNA decreased in SDCBP-depleted CCL-138, CCL-138-R, HTB-43, and HTB-43-R cells. mRNA expression of Nanog, Sox2, and Oct3/4 was found to decrease slightly in the resistant cells derived from all three HNSCC cell lines studied, with the only exception being Sox2 mRNA in JHU029-R cells (Figure 3A). Moreover, CD44 and Sox2 protein levels were reduced upon SDCBP depletion in all cell lines (Figure 3B).

To confirm the ABCB1 downregulation previously observed at the mRNA level, the ABCB1 protein was analyzed by flow cytometry (Figure 3C and Figure S7A). Upon SDCBP depletion, a significant decrease in ABCB1 was observed in all the cell lines (even in CCL-138 cells, not detected at the mRNA level). CCL-138-R and HTB-43-R cells showed higher ABCB1 expression than the parental cells. Overall, the effect of SDCBP inhibition on the sensitization to CDDP was accompanied by a decrease in stemness-related genes in all the HNSCC cell lines assessed.

### 3.5. SDCBP Depletion Induces Apoptosis

To determine whether the role of SDCBP was linked to apoptosis, we performed an Annexin-V/PI analysis. Upon SDCBP depletion, an increase in apoptosis was observed in all three HNSCC cell lines (Figure 3D and Figure S8). Overall, SDCBP inhibition results in a moderate increase in apoptosis in HNSCC cells.

### 3.6. SDCBP Modulates Stemness through p-Src Signaling

**Figure 4:**
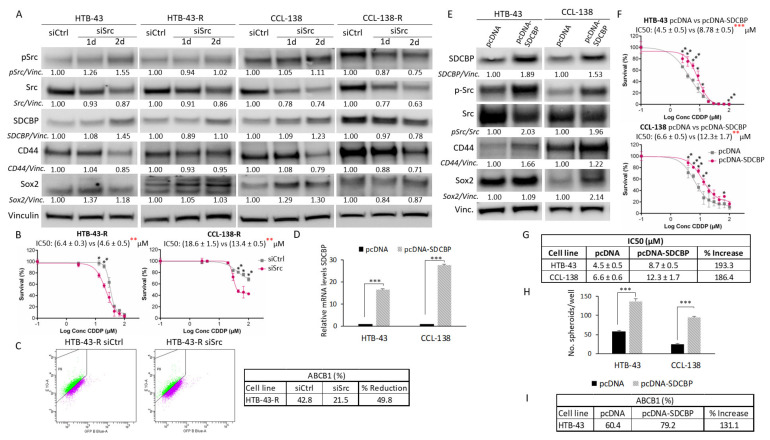
Src inhibition effects and SDCBP overexpression. (**A**) The indicated cell lines were transduced with a siRNA against Src (siSrc) or a scramble siRNA (siCtrl) and examined for Src inhibition at 24 and 48 h post-transfection. Quantification of p-Src/Vinculin, total Src/Vinculin (p-Src/Src ratio is not indicated), SDCBP/Vinculin, CD44/Vinculin, and Sox2/Vinculin expression is indicated. Note the decrease in the Src total levels and p-Src levels in CCL-138-R cells. Note also the decrease in the CD44 protein in all cases; (**B**) Survival curves of cells depleted of Src in response to CDDP. Note that Src inhibition sensitizes HTB-43-R and CCL-138-R cells to CDDP; (**C**) Flow cytometry study indicating HTB-43 cells positive for ABCB1 upon Src inhibition (upper panel). The percentages of each sample and the reduction are indicated in the table (lower panel); (**D**) The indicated cell lines were transduced with pcDNA-SDCBP, and SDCBP RNA expression was evaluated by qRT-PCR at 24 h post-transfection; (**E**) Western blot and quantification of the SDCBP/Vinculin, p-Src/Src ratio, CD44/Vinculin, and Sox2/Vinculin levels in cells from the experiment shown in Figure 4D; (**F**) HTB-43 and CCL-138 cells overexpressing SDCBP were treated with CDDP, and their response was determined via IC50 values. (**G**) Table representing the IC50 values of each condition of the experiment shown in Figure 4F and the percentage of increase; (**H**) Graphs indicating the number of spheres generated from cells overexpressing SDCBP; (**I**) Analysis of ABCB1 expression by flow cytometry of HTB-43 cells overexpressing SDCBP. * *p* < 0.05, ** *p* < 0.01, *** *p* < 0.001.

To define whether SDCBP activates p-Src as described in melanoma [25,26], p-Src expression was analyzed by Western blot. The p-Src levels were found to decrease in CCL-138, CCL-138-R, HTB-43, HTB-43-R, and JHU029-R cells upon SDCBP depletion (Figure 3B). The same trend was observed in biopsy-derived cell lines (Figure 1E). Our results suggest that p-Src inhibition precedes CD44 and Sox2 inhibition in SDCBP-depleted CCL-138-R, HTB-43, and HTB-43-R cells. Moreover, in CCL-138 and CCL-138-R cells, treatment with dasatinib (a major p-Src inhibitor) led to a reduction in the CD44 and Sox2 protein levels (Figure S9A) without affecting the SDCBP expression levels (data not shown), and it sensitized HNSCC cells to CDDP (Figure S9B). To corroborate that the effects of SDCBP are dependent on Src signaling, Src expression was depleted by the siRNA approach. Src knockdown was verified at 24 and 48 h post-transfection (Figure 4A). Although p-Src was not as drastically inhibited by siRNA compared to dasatinib treatment, we observed partial Src inhibition accompanied by CD44 downregulation (Figure 4A). Moreover, p-Src/Src inhibition sensitized HNSCC cells to CDDP and reduced ABCB1 expression (Figure 4B,C). Our results suggest that p-Src is a major downstream player in SDCBP-mediated CSC properties and CDDP resistance.

### 3.7. SDCBP Overexpression Increases CDDP Resistance and Stemness Properties

To corroborate our previous results, we generated SDCBP-overexpressing HNSCC cells using two different plasmids containing SDCBP cDNA to transfect CCL-138 and HTB-43 cells. First, SDCBP overexpression (pcDNA-SDCBP) in HTB-43 and CCL-138 cells was demonstrated at the RNA and protein levels (Figure 4D,E). According to the drug sensitivity assays, SDCBP overexpression led to increased IC50 values for CDDP in the HTB-43 and CCL-138 cells compared to the control cells (Figure 4F,G). In addition, SDCBP-overexpressing HNSCC cells also showed an increased tumorsphere formation capacity (Figure 4H). Moreover, p-Src, Sox2, CD44, and ABCB1 protein levels increased in SDCBP-overexpressing HNSCC cells, consistent with the observed effects of SDCBP depletion (Figure 4E–I and Figure S7B). Second, to observe a gradual increase in SDCBP expression over time, HTB-43 cells were transfected with the pIRES2-EGFP-SDCBP plasmid and selected with the G418 antibiotic to obtain a culture that stably overexpressed SDCBP. SDCBP expression increased at both the RNA and protein levels, reaching higher overexpression levels with respect to the pcDNA plasmid (Figure S10A,B). CD44 and Sox2 increased with a delay in relation to p-Src activation (Figure S10B). qRT-PCR analysis revealed that CD44, ABCB1, and KLF4 mRNA levels were increased at 24 or 48 h after pIRES2-EGFP-SDCBP transfection compared with control cells (Figure S10C). Our findings support our previous findings indicating that SDCBP expression is involved in the modulation of CSC properties and CDDP resistance, with Src activation emerging as a main downstream target.

### 3.8. SDCBP Inhibition Reduces Tumor Formation Capacity in Mice

**Figure 5:**
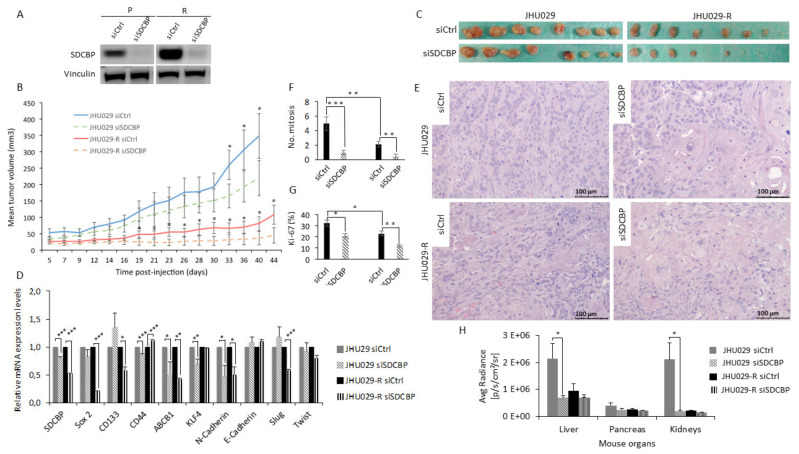
SDCBP inhibition reduced tumor growth. (**A**) Western blot of JHU029 and JHU029-R cells depleted of SDCBP injected into the mice. The cells were maintained in culture for up to 72 h to evaluate SDCBP inhibition; (**B**) The mean tumor volume of the tumors formed by JHU029 and JHU029-R cells over the duration of the experiment; (**C**) Pictures of tumors at the endpoint of the experiment; (**D**) qRT-PCR analysis of the mRNA expression of the indicated genes in the tumors. SDCBP is still inhibited at the endpoint of the experiment compared with control tumors; (**E**) Morphological aspects of tumors formed by the indicated types of cells (Hematoxylin and eosin staining). SDCBP reverses the fusocellular pattern of tumors induced by JHU029-R cells; (**F**) Number of mitoses per 3 randomly selected microscopic fields/tumor (40×) in the indicated types of tumors (see Figure S12A); (**G**) Number of Ki-67-positive cells in 3 independents randomly selected microscopic fields/tumor in the indicated types of tumors (see Figure S12B); (**H**) Graph of the mean radiance in the indicated organs indicative of metastatic cells. * *p* < 0.05, ** *p* < 0.01, *** *p* < 0.001.

JHU029 cells were chosen because they express high levels of the SDCBP protein (Figure S4E). SDCBP depletion was confirmed by Western blot prior to injection in the mice (Figure 5A). We observed that the tumors formed by SDCBP-depleted cells grew more slowly than those formed by the corresponding control cells (Figure 5B). At the experimental endpoint, we also verified that SDCBP-depleted tumors were smaller than the tumors in the control groups (Figure 5C). The tumors formed by JHU029-R cells showed slower tumor growth rates than the tumors formed by JHU029 cells, as previously observed for the HTB-43 and CCL-138 cell lines (Figure S3A) and reported by us for JHU029 cells [14]. RNA was extracted from two xenografted tumors randomly selected from each group: JHU029 siCtrl, JHU029 siSDCBP, JHU029-R siCtrl, and JHU029-R siSDCBP. qRT-PCR analysis revealed that SDCBP depletion was concomitantly accompanied by a reduced expression in CSC-related genes (Figure 5D), corroborating our previous in vitro data shown in Figure 3A. The pathological analysis of xenografted tumors revealed that: (i) tumors formed by SDCBP-depleted JHU029-R cells exhibited an epithelioid pattern that was markedly different from that of tumors formed by JHU029-R cells (Figure 5E); (ii) tumors harboring SDCBP depletion showed less mitosis; and (iii) they showed a lower Ki-67 proliferation index than their control cells (Figure 5F,G and Figure S11A,B). Decreases in N-cadherin and Slug mRNA were detected in SDCBP-depleted JHU029-R cells, suggesting the involvement of EMT modulation, in addition to the decrease in Sox2 and ABCB1. Some mouse organs were analyzed by bioluminescence to detect the presence of metastases. SCDBP depletion decreased the metastatic ability of JHU029 cells (Figure 5H and Figure S12). Furthermore, SDCBP inhibition, by modulating stemness, was able to revert the fusocellular pattern of tumors formed by JHU029-R cells, suggesting a role of SDCBP in the EMT program in vivo. Overall, these findings demonstrate that the oncogenic role of SDCBP in HNSCC involves the modulation of stemness, tumor aggressiveness, and metastatic potential, probably through EMT. In addition, we showed that SDCBP depletion effectively counteracted tumorigenesis and metastatic dissemination in vivo in both CDDP-resistant and -nonresistant HNSCC models.

### 3.9. SDCBP Expression in HNSCC Patients

**Figure 6:**
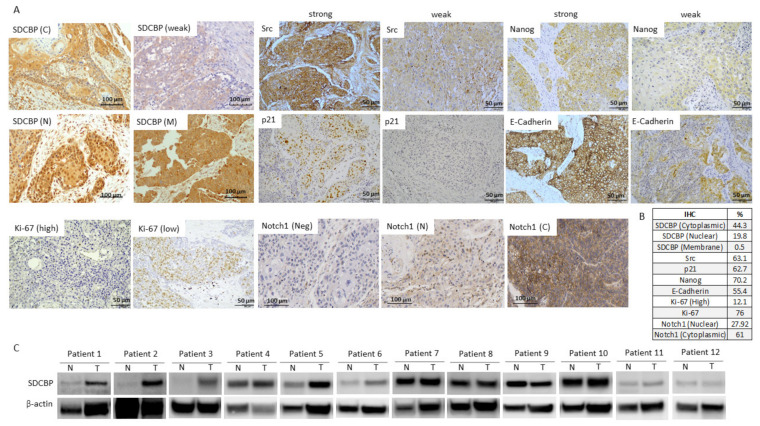
SDCBP was overexpressed in cancer tissue samples from a cohort of 382 patients. (**A**) Representative IHC pictures showing the expression of SDCBP, active Src, p21, Nanog, E-cadherin, ki-67, and Notch1 in HNSCC patients; (**B**) Percentage of positive patients (considering the 382 patients analyzed) to each of the following proteins: SDCBP, active Src, p21, Nanog, E-cadherin, Ki-67, and Notch1; (**C**) Western blot of the SDCBP protein levels in normal and tumoral tissue samples of 12 patients. C, cytoplasmic; M, membrane; N, nuclear; Neg, Negative expression.

Next, the clinical relevance of SDCBP was explored in a cohort of 382 HNSCC patients. The IHC analysis of SDCBP, Ki-67, active Src (p-Src), E-cadherin, Nanog, Notch1, and p21 was performed in HNSCC tissue specimens. SDCBP upregulation was frequently detected, showing both cytoplasmic (44.3%) and nuclear (19.8%) patterns and rare membrane localization (Figure 6A,B and Figure S13A). E-cadherin was found in the membrane; Nanog was found in the cytoplasm; and Ki-67, active Src, p21, and Notch1 were found in the nucleus at the indicated percentages (Figure 6C).

**Figure 7:**
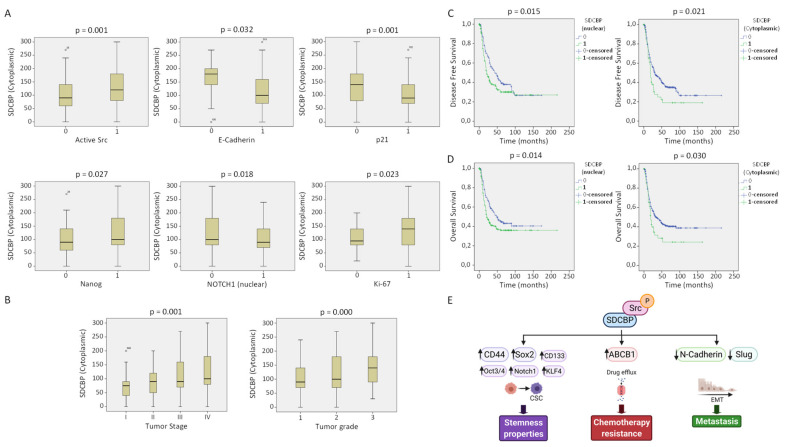
SDCBP correlation with clinical parameters and proteins. (**A**) Direct correlation of cytoplasmic SDCBP expression with the presence of active Src (p-Src), Nanog (nuclear), and Ki-67. Inverse correlation of cytoplasmic SDCBP expression with the epithelial marker E-cadherin, tumor suppressor p21, and Notch1 (nuclear); (**B**) High SDCBP expression is associated with more aggressive tumors (tumor grade) and more advanced tumors (tumor stage); (**C**) Direct association of disease-free survival and SDCBP expression in the cytoplasm and nucleus; (**D**) Direct association of overall survival and SDCBP expression in the cytoplasm and nucleus; (**E**) Proposed model by which SDCBP acts as an oncogenic protein in vitro and in vivo (figure created with Biorender).

The SDCBP protein levels were positively associated with Src activation (*p* = 0.001) and Nanog and Ki-67 expression but inversely associated with E-cadherin (*p* = 0.032), Notch1 (*p* = 0.018), and p21 (*p* = 0.001) expression (Figure 7A). This outcome is in good agreement with the mouse experiments showing EMT features associated with SDCBP expression that reverted to a more epithelioid pattern after SDCBP silencing, suggesting a role of SDCBP in EMT. The inverse correlation between the expression of SDCBP and the tumor suppressors p21 and Notch1 supports the oncogenic role of SDCBP in HNSCC (Figure 7A). SDCBP expression was corroborated by Western blot in paired normal and tumor tissue samples from 12 HNSCC patients. Six of 12 patients exhibited SDCBP upregulation in the tumor tissues compared to the patient-matched normal tissues, corroborating the importance of the frequent aberrant expression of SDCBP in HNSCC (Figure 6C). SDCBP expression was also associated significantly with advanced tumor stage and poor tumor grade (Figure 7B). Moreover, cytoplasmic, nuclear, and membrane SDCBP expression levels were significantly associated with shorter disease-free survival and overall survival, supporting the SDCBP mRNA levels reported in TCGA database (Figure 7C,D and Figure S13A–C).

## 4. Discussion

In the present study, a comparative proteomic analysis identified SDCBP as a protein that is upregulated in CDDP-resistant HNSCC cells and CSCs. Aberrant expression of SDCBP in some cancers has been associated with tumor progression, metastatic dissemination, and poor prognosis via FAK, Src, p38-MAPK, AKT, NFκB, IGFBP2, EGFR, and VEGFR [9,27,28,29,30]. On this basis, we aimed to investigate the role of SDCBP in HNSCC chemoresistance in vitro and in vivo. We demonstrated that SDCBP depletion modulates CSC properties and CDDP resistance in the different HNSCC cell lines tested. Tumorsphere formation capacity was markedly reduced upon SDCBP depletion in both parental and CDDP-resistant HNSCC cells.

In the present article, we found that SDCBP is involved in CDDP resistance, representing a novel aspect of the multifaceted role of SDCBP in tumor biology. In fact, only two recent studies associated SDCBP with drug resistance in prostate and colon cancer [31,32]. The role of SDCBP in resistance observed in vitro is further supported by tumors generated in vivo harboring consistently higher protein levels of SDCBP than sensitive tumors. CSCs and CDDP-resistant HNSCC cells tumors grew slowly and showed higher tumorigenic potential by increasing: (i) tumorsphere-forming ability and CSC properties; (ii) Ki-67-positive cell numbers; (iii) tumor heterogeneity; and (iv) metastatic ability (only for CSCs) compared to parental cells. This finding supports previous reports, including ours, showing that resistant cells (and CSCs) have higher CSC gene expression and are less proliferative [14,23,33,34]. Importantly, the fact that SDCBP overexpression with the two different constructs has an impact directly on resistance acquisition without hardly affecting cell proliferation (data not shown) corroborates the direct effect previously observed of SDCBP on resistance. Moreover, the fact that SDCBP overexpression increases stemness properties and ABCB1 expression confirms such a statement. Lastly, Src inhibition—the direct downstream target of SDCBP—is able to reduce resistance and ABCB1 expression. Overall, the experiments demonstrate that SDCBP is directly involved in the acquisition of resistance in primary and established head and neck cancer cell lines.

Our results showed that SDCBP depletion led to a reduction in the CSC-related genes CD44, Sox2, Nanog, Oct3/4, CD133, KLF4, and ABCB1 (CD44, Sox2, and ABCB1 were also confirmed at the protein level). The reduction in CSC marker expression in SDCBP-depleted cells was particularly remarkable in CDDP-resistant cells. The biological function of SDCBP in HNSCC cells is also supported by SDCBP overexpression experiments, further confirming a key role of SDCBP in the modulation of stemness and chemoresistance. Our results corroborate the role of SDCBP in CSCs, as reported in prostate cancer [31].

Conversely, it was suggested that SDCBP binds to Src in melanoma, resulting in its activation (p-Src) and downstream signaling [9]. We found that the Src blockade inhibits SDCBP-mediated resistance. Our data extrapolate SDCBP-Src signaling to the HNSCC model and extend the action of SDCBP to the modulation of stemness and CSC gene expression (CD44, Sox2, and ABCB1).

To the best of our knowledge, this study was the first to target SDCBP expression and function in an in vivo laryngeal cancer model specifically. SDCBP inhibition significantly reduced tumor growth in immunosuppressed mice. Interestingly, the tumors formed by SDCBP-depleted cells were smaller in size and harbored a lower mitotic index and Ki-67 positivity rate than tumors formed by control cells. In addition, SDCBP depletion reverted the fusocellular pattern exhibited by JHU029-R cells to a more epithelial phenotype, thereby revealing a possible link between SDCBP and EMT in HNSCC. The role of SDCBP as an EMT inducer in breast cancer and melanoma has been associated with small GTPases and Slug, respectively [35,36]. In fact, we found a marked reduction in the mRNA levels of N-cadherin and Slug in SDCBP-depleted JHU029-R cells. Moreover, the role of SDCBP in metastatic dissemination was shown in SDCBP-depleted JHU029 xenografted tumors. This finding could be explained by JHU029-R tumors exhibiting EMT features, while JHU029 tumors proliferate more rapidly (higher Ki-67-positive cells and higher mitosis) than JHU029-R tumors.

The clinical and pathological relevance of SDCBP was further explored by IHC analysis in a large cohort of HNSCC patients. SDCBP expression in HNSCC patient biopsies was significantly associated with the expression of active Src, Nanog, and Ki-67 and inversely associated with E-cadherin, p21, and Notch1. Our results confirmed the tumor-suppressive role of the Notch pathway in HNSCC, as proposed by other authors [37,38]. These findings provide strong support for our in vitro data, indicating the oncogenic role of SDCBP in HNSCC and its contribution to CSC maintenance and EMT through Src signaling activation (Figure 7E). Importantly, SDCBP protein expression had a major impact on HNSCC patient prognosis and was significantly associated with poor clinical outcomes and more aggressive phenotypes.

## 5. Conclusions

Our findings reveal the clinical relevance and oncogenic function of SDCBP in HNSCC. The evaluation of both patient biopsies and preclinical models demonstrated the involvement of SDCBP in chemoresistance, stemness, and metastasis. Resistant cells and CSCs represent the main determinants of therapy resistance and recurrence; therefore, it is of paramount importance to develop therapeutic strategies against such cellular populations. Since the use of Src or ABCB1 inhibitors has shown modest results in the clinic due to their high toxicity [39,40], the targeting of SDCBP could be a more effective therapeutic strategy to eliminate resistant tumors in HNSCC patients effectively.

## Data Availability

The data presented in this study are available on request from the corresponding author. Table S1 has been deposited to the Figshare as: 10.6084/m9.figshare.13614449. The mass spectrometry proteomics data (the 36 most important deregulated proteins are shown in the Figure S1) were deposited to the ProteomeXchange Consortium via the PRIDE partner repository, dataset identifier PXD020159. More information about this proteomic analysis can be found in Tables S2–S5 of our previous report [14].

## Supplementary Materials

The following are available online at https://www.mdpi.com/article/10.3390/cancers13194952/s1, Table S1 (Available in the repository data Figshare as: 10.6084/m9.figshare.13614449.): Clinical and pathological characteristics of the 382 HNSCC patients analyzed by IHC, Figure S1: Scheme of the proteomic study and the resulting proteins; Figure S2: Acquisition of CDDP resistance in the HTB-43 and CCL-138 cell lines in vivo; Figure S3: Mouse tumors formed by parental cells, resistant cells, and CSCs; Figure S4: SDCBP inhibition; Figure S5: SDCBP inhibition with an independent siRNA; Figure S6: SDCBP inhibition is maintained over time; Figure S7: ABCB1 analysis; Figure S8: Analysis of apoptosis by flow cytometry; Figure S9: Effect of dasatinib treatment in the CCL-138 cell line; Figure S10: HTB-43 cells overexpressing pIRES2-EGFP-SDCBP; Figure S11: Mouse tumors formed by JHU029 and JHU029-R cells depleted of SDCBP; Figure S12: Metastasis in mice; Figure S13: SDCBP and clinical parameters.
