# Supplementary material for: SDCBP Modulates Stemness and Chemoresistance in Head and Neck Squamous Cell Carcinoma through Src Activation
Source: Cancers (Basel). 2021 Oct 1;13(19):4952. doi: 10.3390/cancers13194952 (PMC8508472; doi:10.3390/cancers13194952)
Supplement: Supplementary file 1 [file cancers-13-04952-s001.zip › Suppl. Table and figures 11-08-21.pdf]

Supplementary tables and figures

Supplementary Tables

Table S1. Clinical and pathological characteristics of the 382 HNSCC patients analyzed by IHC. Available in the repository data Figshare as: 10.6084/m9.figshare.13614449.

Supplementary Figures

Figure S1. Scheme of the proteomic study and the resulting proteins. Upregulated and downregulated proteins, identified by the comparative proteomic analysis of CCL-138 parental cells (P), CDDP-resistant cells (R), and cancer stem cells (CSCs), are shown.

Figure S2. Acquisition of CDDP resistance in the HTB-43 and CCL-138 cell lines *in vivo*. The mean tumor volumes of mouse tumors (control versus CDDP-treated tumors) formed by the indicated cell lines are presented. (**A**) The mean tumor volume of HTB-43 control versus CDDP-treated tumors on the indicated days. (**B**) Survival curves of representative examples of control and resistant tumors explanted *in vitro* to determine their response to CDDP for the indicated types of tumors formed by HTB-43 cells. Log CONC represent CDDP concentration in a logarithmic scale (**C**) The mean tumor volume of CCL-138 control versus treated tumors. (**D**) Survival curves of representative examples of control and resistant tumors explanted *in vitro* to determine their response to CDDP for the indicated tumor types formed by CCL-138 cells. Log CONC represent CDDP concentration in a logarithmic scale.

Figure S3. Mouse tumors formed by parental cells, resistant cells, and CSCs. (**A**) The mean tumor volume of parental cells, CDDP-resistant cells, and cancer stem cells of the

HTB-43 cell line (left panel). The mean tumor volume of parental cells, CDDP-resistant cells, and cancer stem cells of the CCL-138 cell line (right panel). (**B**) Representative images of HTB-43 cells, HTB-43-R cells, and CSCs (derived from HTB-43 cells) positive for Ki-67. Note that there were more Ki-67-positive HTB-43-R cells and CSCs than HTB-43 control cells. (**C**) Morphological aspects of tumors formed by CCL-138 cells, CCL-138-R cells, and CSCs (derived from CCL-138 cells) (H&E staining). Note that tumors formed by CCL-138 cells are more differentiated than those formed by CCL-138-R and CSCs. (D) H&E and p63 staining indicating the presence of squamous carcinoma cells from human cells in the lungs of mice injected with HTB-43 cells and HTB-43 CSCs.

Figure S4. SDCBP inhibition. (**A**) SDCBP expression by western blot in SCC-25 cells depleted of SDCBP. (**B**) Survival curves indicating the IC50 values of SDCBP-depleted cells in response to CDDP (the plots correspond to the data summarized in Figure 1D). Log CONC represent CDDP concentration in a logarithmic scale. (**C**) Survival curves indicating the IC50 values of SDCBP-depleted SCC-25 cells in response to CDDP. Log CONC represent CDDP concentration in a logarithmic scale. (**D**) IC50 values from the experiment from Figure S4C indicating the IC50 reduction. (**E**) SDCBP protein expression by western blot in the different HNSCC cell lines.

Figure S5. SDCBP inhibition with an independent siRNA. (**A**) Western blot of CCL-138, CCL-138-R, HTB-43, and HTB-43-R cells depleted for SDCBP using a different siRNA than those used in Figure 1A (designated 2siSDCBP) versus their corresponding control (2siCtrl). (**B**) Survival curve of HTB-43 cells depleted with 2siSDCBP in response to CDDP. Log CONC represent CDDP concentration in a logarithmic scale. (**C**)

Corresponding IC50 values for Figure S5B and the corresponding percentage of reduction. (**D**) Spheroid area of the indicated cell lines. *$^{**}p < 0.01$, $^{***}p < 0.001$.*

Figure S6. SDCBP inhibition is maintained over time. (**A**) Western blot of SDCBP inhibition in the indicated HNSCC parental and resistant cells depleted of SDCBP. (**B**) Images of spheres from 3D culture (100X) of the indicated cell lines from the experiment shown in Figure 2A.

Figure S7. ABCB1 analysis. (**A**) Flow cytometry plots of ABCB1 expression in CCL-138, CCL-138-R, HTB-43, HTB-43-R, JHU029, and JHU029-R cells depleted of SDCBP. The plots correspond to the experiment shown in Figure 3C. (**B**) Plots indicating ABCB1 expression in HTB-43 cells overexpressing SDCBP (pcDNA-SDCBP) versus HTB-43 control cells (pcDNA).

Figure S8. Analysis of apoptosis by flow cytometry. The indicated HNSCC cells were depleted of SDCBP, and apoptosis induction was analyzed. PI corresponds to A-BlueA fluorescence and Annexin V-APC to C Red-A fluorescence. Early apoptosis (LR) and late apoptosis (UR) percentages are indicated in each plot of the indicated cell lines. These results are summarized in Figure 3B. The results from one experiment are shown and are representative of three independent experiments. *LR: lower right; UR: upper right*.

Figure S9. Effect of dasatinib treatment in the CCL-138 cell line. (**A**) Western blot analysis of SDCBP, the p-Src/Src ratio, and the CD44 and Sox2 levels in treated cells. (**B**) Effects of dasatinib, CDDP, and the combination of both drugs on the indicated cell lines. The concentrations used for each drug are indicated. *$^{*}p < 0.05$, $^{***}p < 0.001$.*

Figure S10. HTB-43 cells overexpressing pIRES2-EGFP-SDCBP. (**A**) mRNA expression of SDCBP in cells transduced with pIRES2-EGFP-SDCBP was analyzed by qRT-PCR at 24 and 48 hours post-transfection. (**B**) Western blot of the SDCBP, p-Src/Src, CD44, and Sox2 levels in cells overexpressing pIRES2-EGFP-SDCBP versus control cells. (**C**) qRT-PCR results of the SDCBP levels and genes associated with stemness and resistance at days 1 and 2 post-transfection. *Notes:* * $p < 0.05$, ** $p < 0.01$, *** $p < 0.001$.

Figure S11. Mouse tumors formed by JHU029 and JHU029-R cells depleted of SDCBP. (**A**) Representative images of H&E staining in the indicated group of tumors showing examples of mitotic cells. Graphs are shown in Figure 5F. (**B**) Representative images of tumors in the indicated group showing cells positive for the Ki-67 proliferative marker. The results are presented in Figure 5G.

Figure S12. Metastasis in mice. Luminescence images of the different mouse organs for the detection of tumoral cells by the IVIS system.

Figure S13. SDCBP and clinical parameters. SDCBP expression located at the plasma membrane is positively associated with disease-free survival and overall survival (**A**). p values are indicated in each case. (**B**) SDCBP mRNA levels in tumor versus normal tissue (according to TCGA database, from the Human Protein Atlas website (http://www.proteinatlas.org). (**C**) SDCBP mRNA levels in relation to overall survival (according to TCGA database).
