# Supplementary material for: SDCBP Modulates Stemness and Chemoresistance in Head and Neck Squamous Cell Carcinoma through Src Activation
Source: Cancers (Basel). 2021 Oct 1;13(19):4952. doi: 10.3390/cancers13194952 (PMC8508472; doi:10.3390/cancers13194952)
Supplement: Supplementary file 1 [file cancers-13-04952-s001.zip › Supplementary Figure 11 19-07-21.pdf]

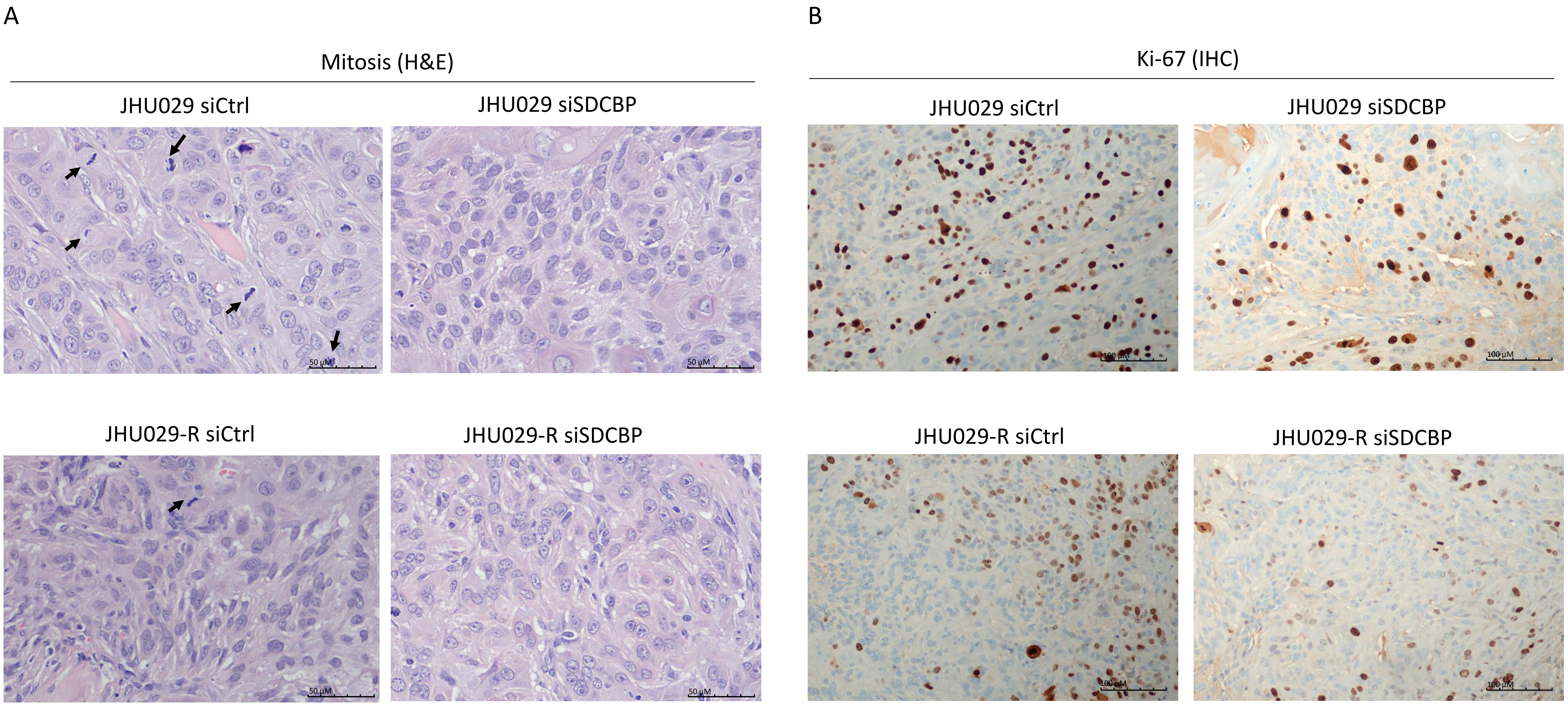
A
Mitosis (H&E)
JHU029 siCtrl
JHU029 siSDCBP
JHU029-R siCtrl
JHU029-R siSDCBP
50 µM
B
Ki-67 (IHC)
JHU029 siCtrl
JHU029 siSDCBP
JHU029-R siCtrl
JHU029-R siSDCBP
100 µM

Figure S11
