# Supplementary material for: SDCBP Modulates Stemness and Chemoresistance in Head and Neck Squamous Cell Carcinoma through Src Activation
Source: Cancers (Basel). 2021 Oct 1;13(19):4952. doi: 10.3390/cancers13194952 (PMC8508472; doi:10.3390/cancers13194952)
Supplement: Supplementary file 1 [file cancers-13-04952-s001.zip › Supplementary Figure 13 19-07-21.pdf]

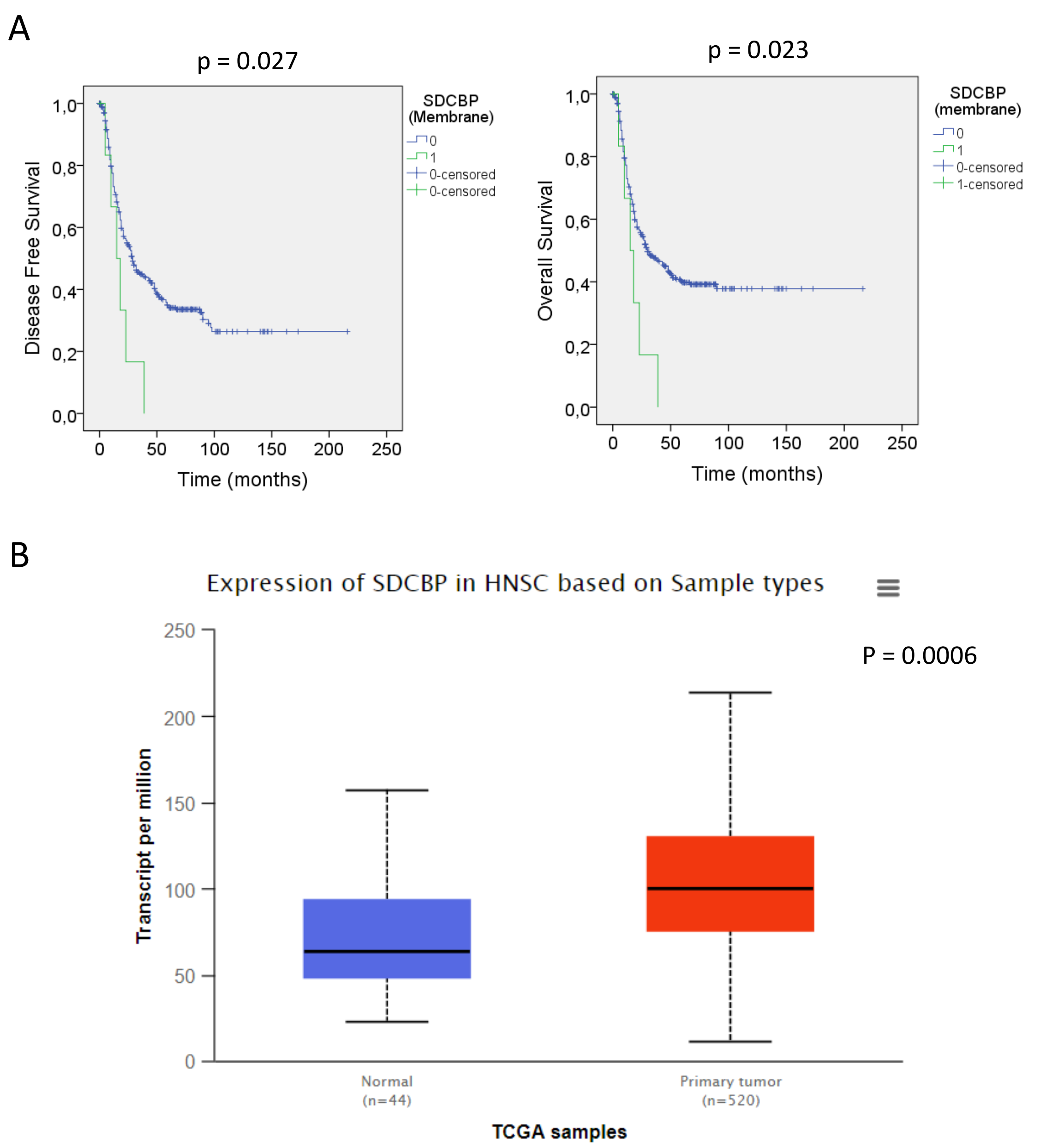

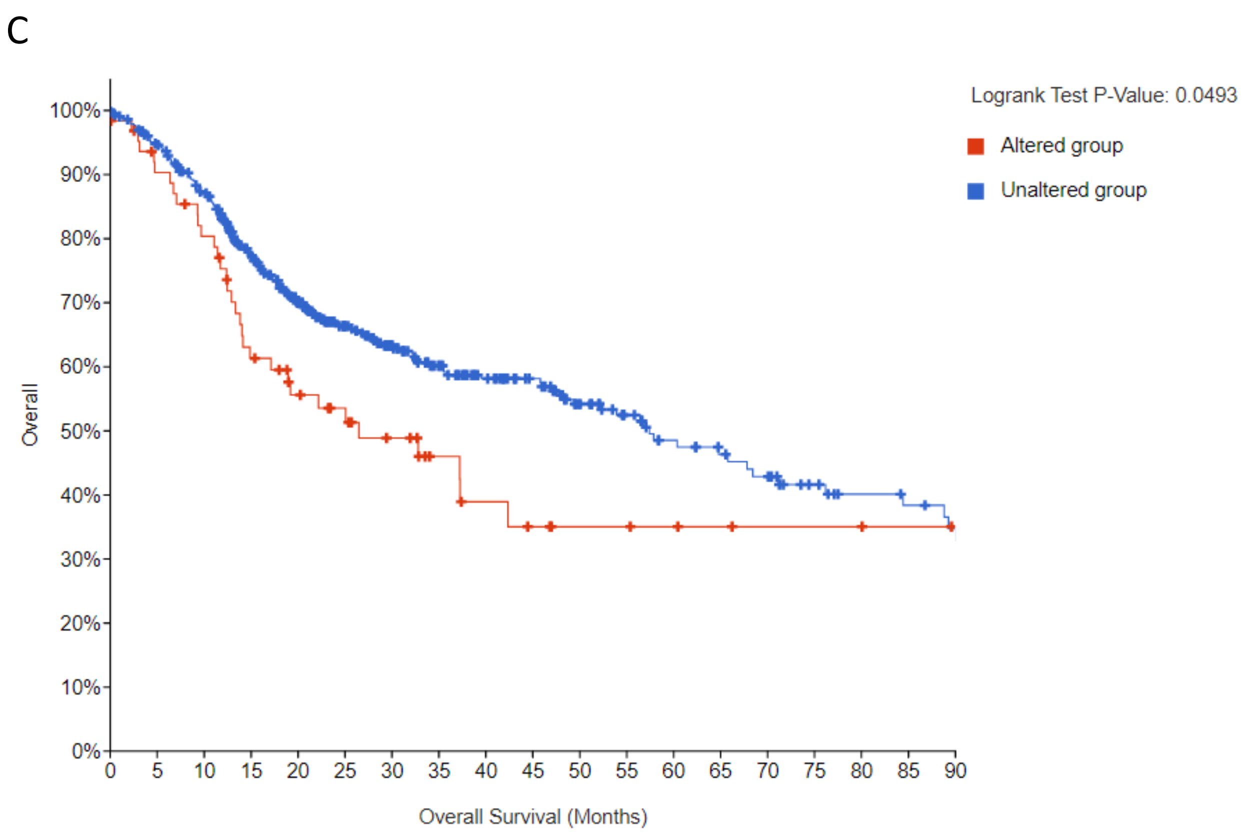

	Number of Cases, Total	Number of Cases, Deceased	Median Months Overall
Altered group	**63**	**33**	**26.48**
Unaltered group	**431**	**179**	**57.42**

Figure S13
