# Supplementary figures and images for: SDCBP Modulates Stemness and Chemoresistance in Head and Neck Squamous Cell Carcinoma through Src Activation
Source: Cancers (Basel). 2021 Oct 1;13(19):4952. doi: 10.3390/cancers13194952 (PMC8508472; doi:10.3390/cancers13194952)

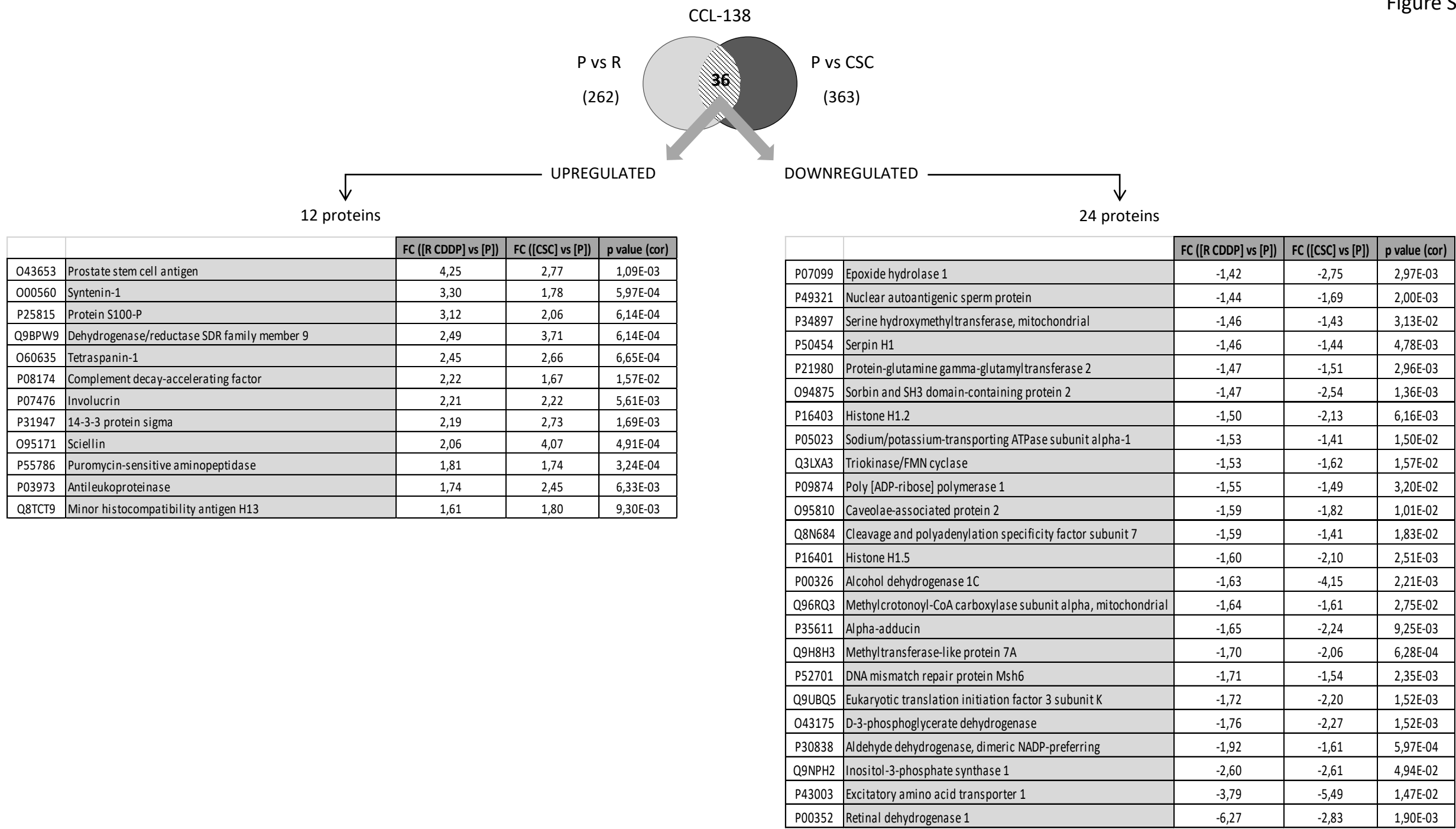

Supplement: Supplementary file 1 [file cancers-13-04952-s001.zip › Supplementary Figure 1 19-07-21.pdf]

A

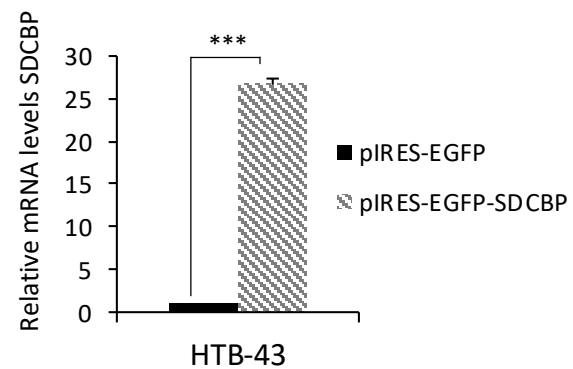

B

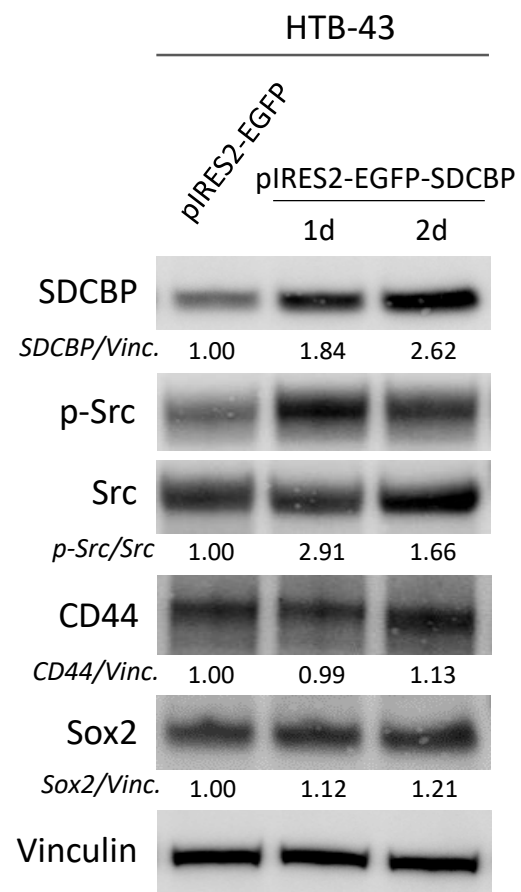

C

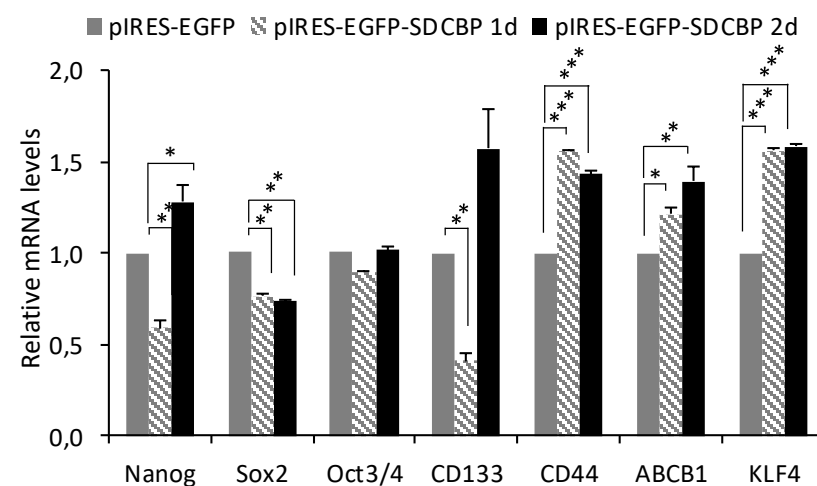

Figure S10

Supplement: Supplementary file 1 [file cancers-13-04952-s001.zip › Supplementary Figure 10 27-08-21.pdf]

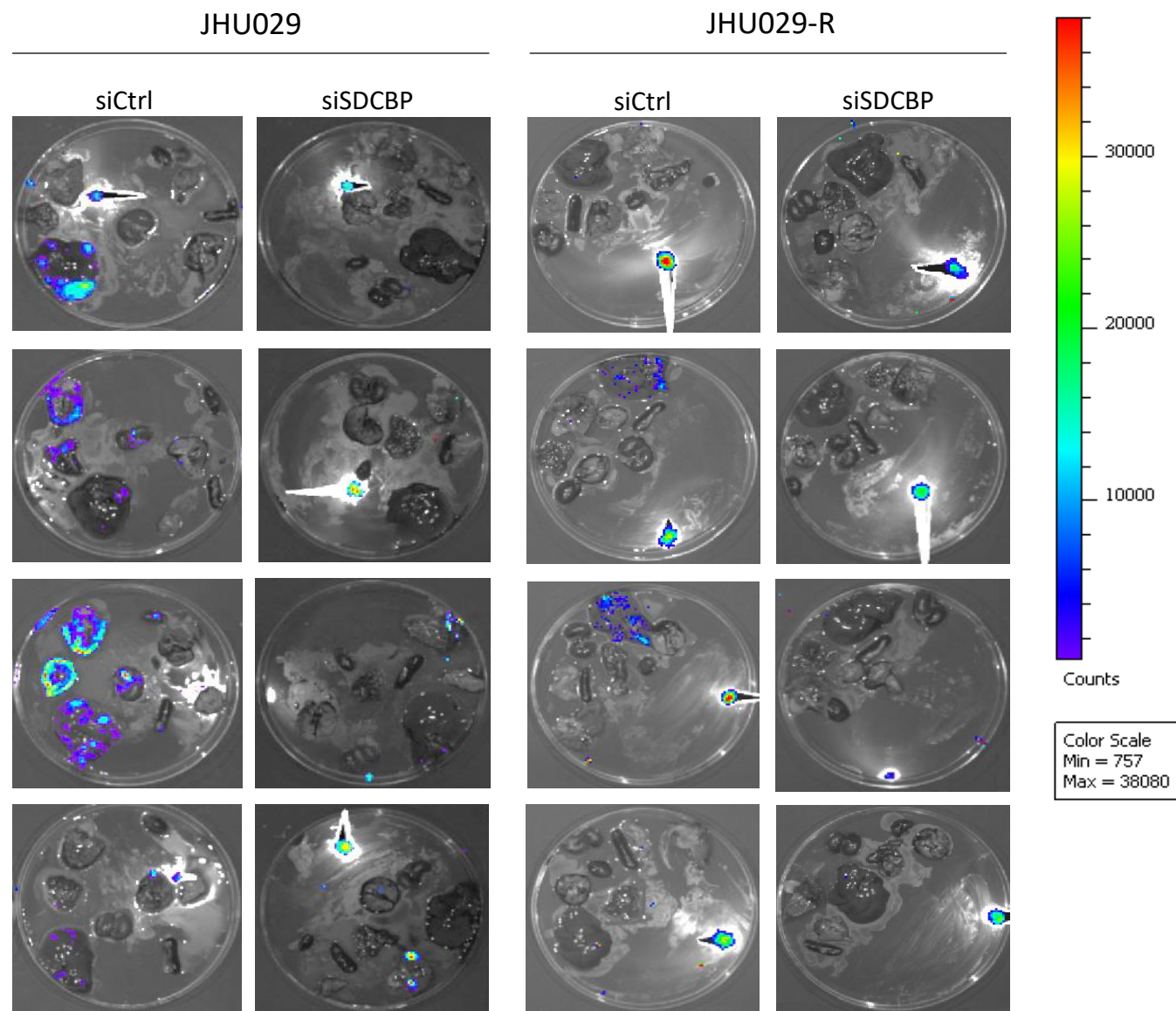

Figure S12

Supplement: Supplementary file 1 [file cancers-13-04952-s001.zip › Supplementary Figure 12 19-07-21.pdf]

A

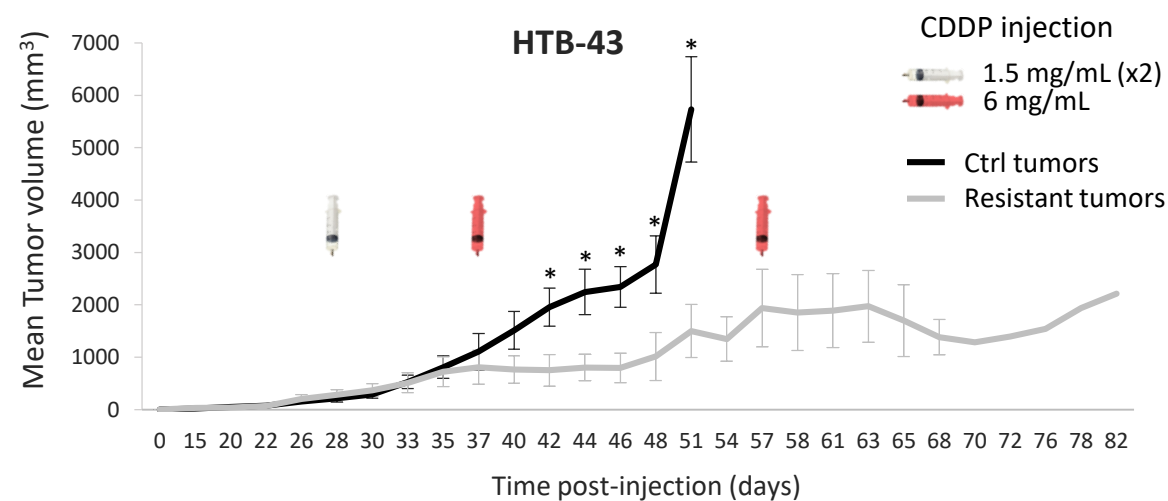

B

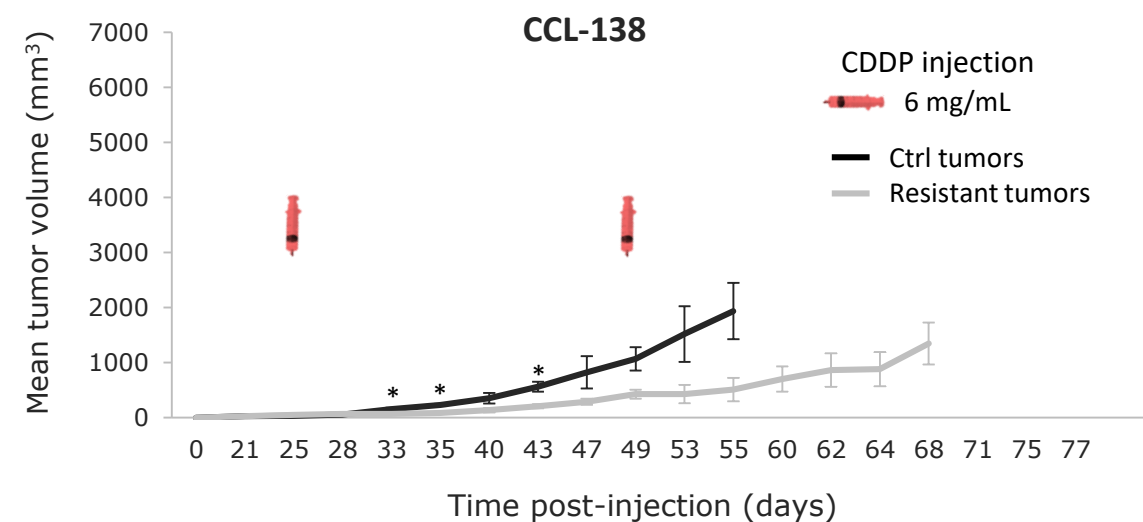

C

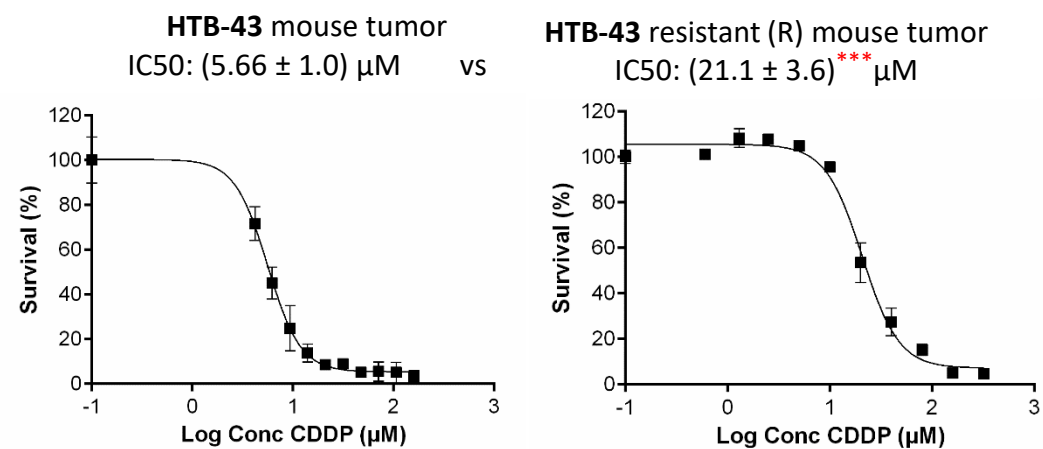

D

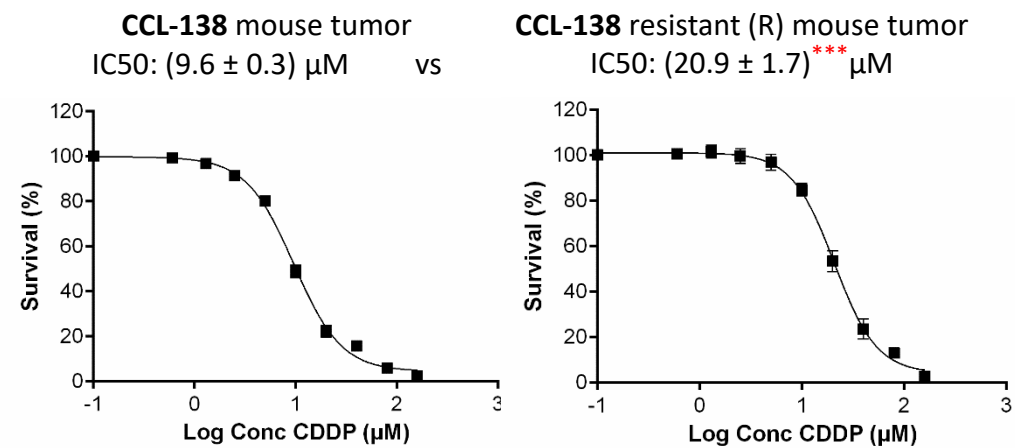

Supplement: Supplementary file 1 [file cancers-13-04952-s001.zip › Supplementary Figure 2 07-09-21.pdf]

A

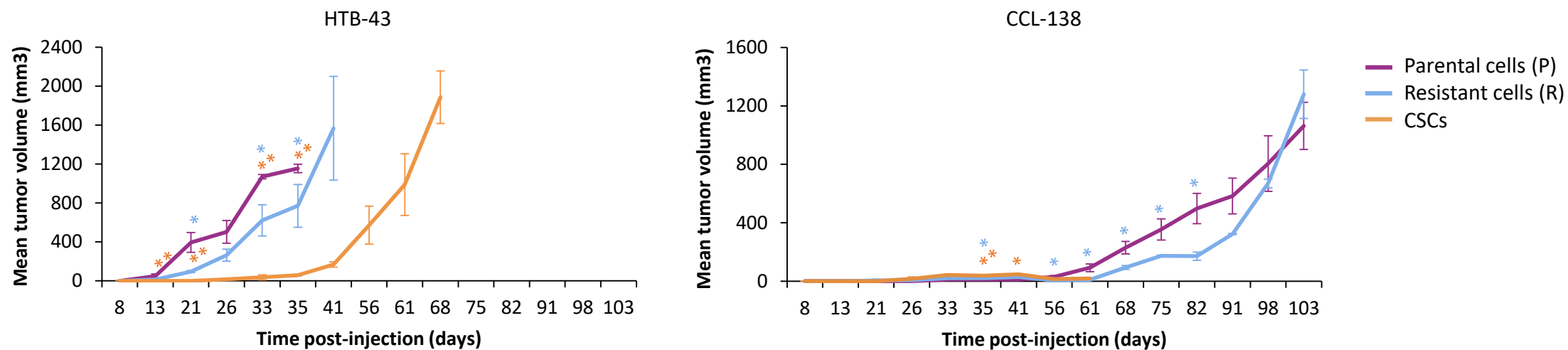

B

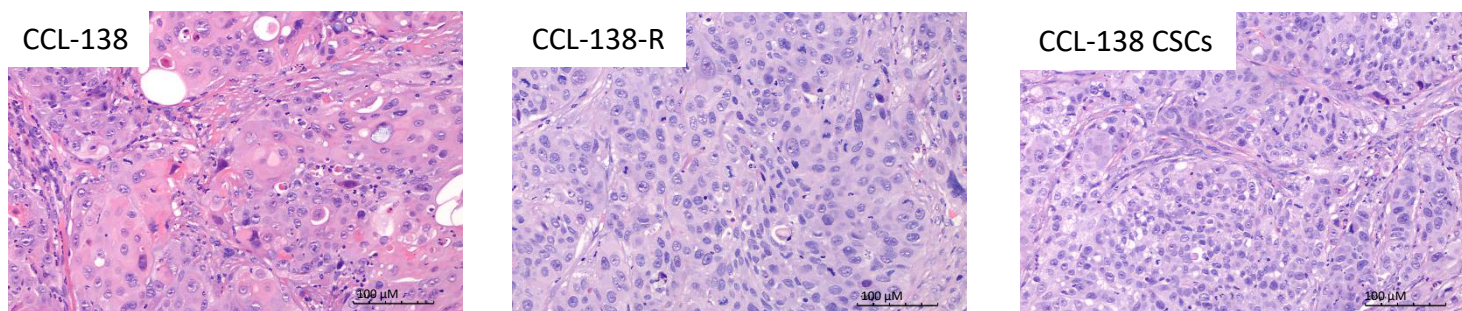

C

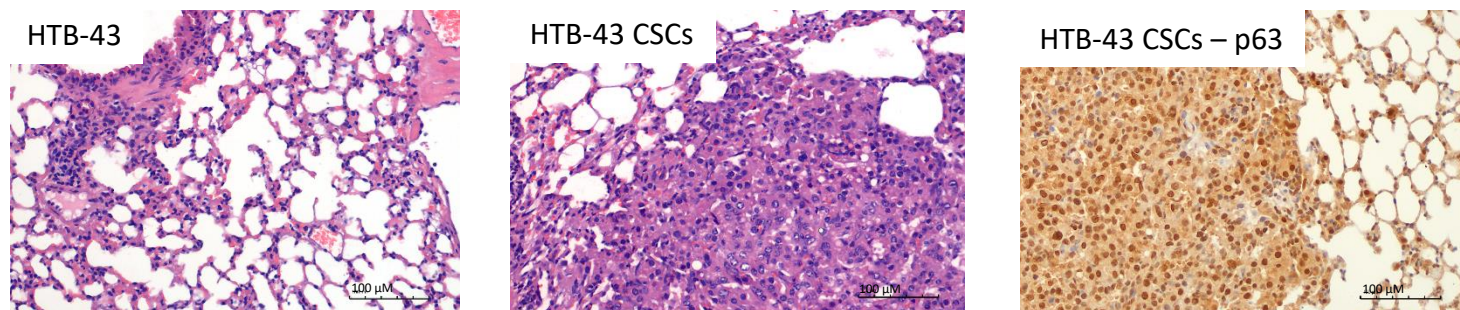

Figure S3

Supplement: Supplementary file 1 [file cancers-13-04952-s001.zip › Supplementary Figure 3 01-09-21.pdf]

A

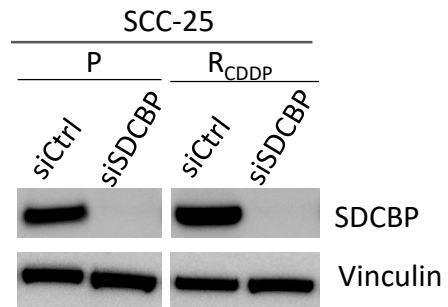

B

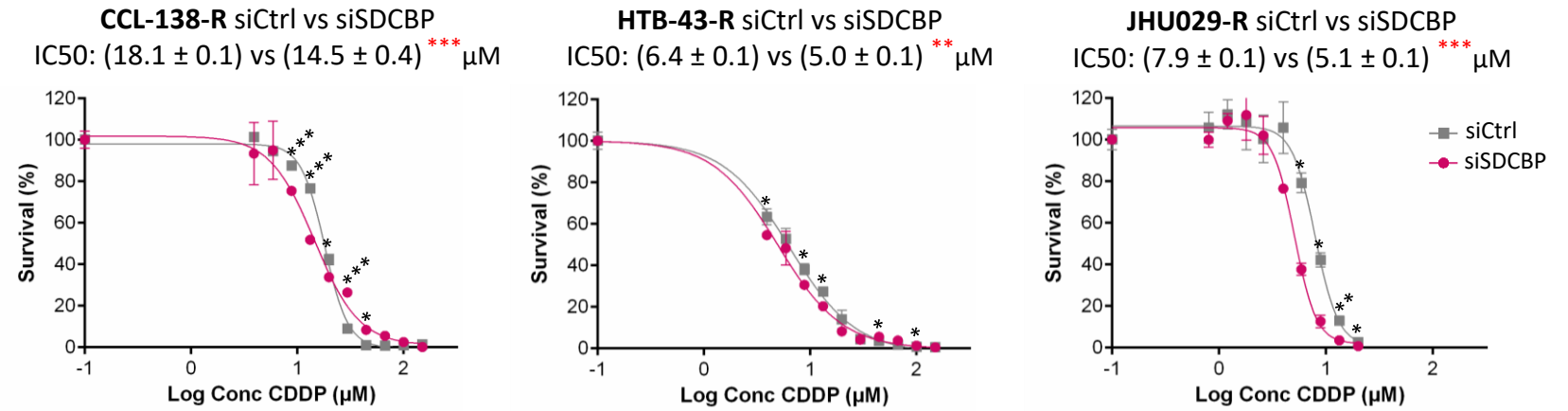

C

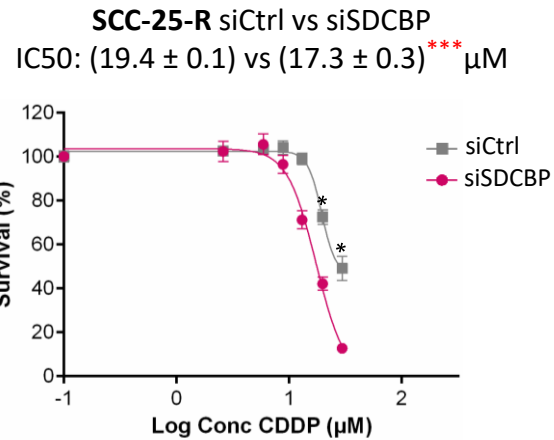

D

| Cell line | IC50 (μM)  |            |             |
|-----------|------------|------------|-------------|
|           | siCtrl     | siSDCBP    | % Reduction |
| SCC-25-R  | 19.4 ± 0.1 | 17.3 ± 0.3 | 10.8        |

E

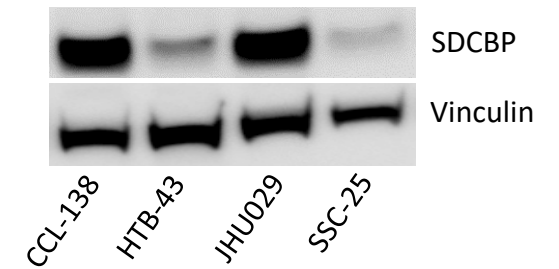

Figure S4

Supplement: Supplementary file 1 [file cancers-13-04952-s001.zip › Supplementary Figure 4 07-09-21.pdf]

A

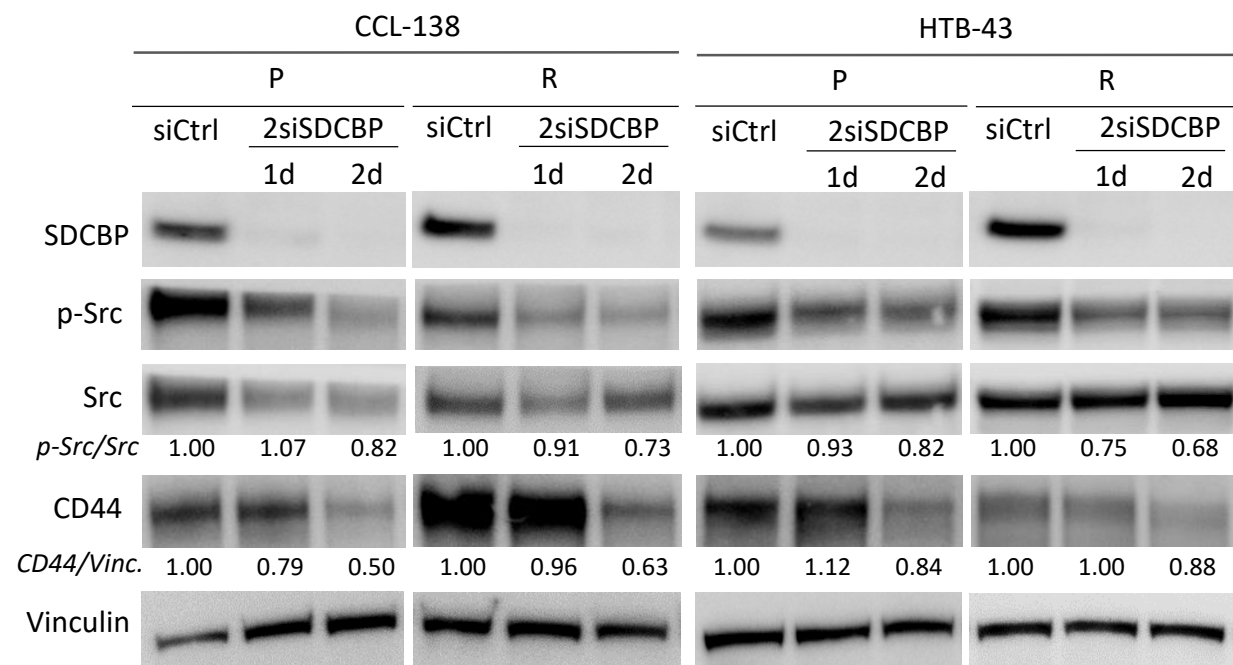

B

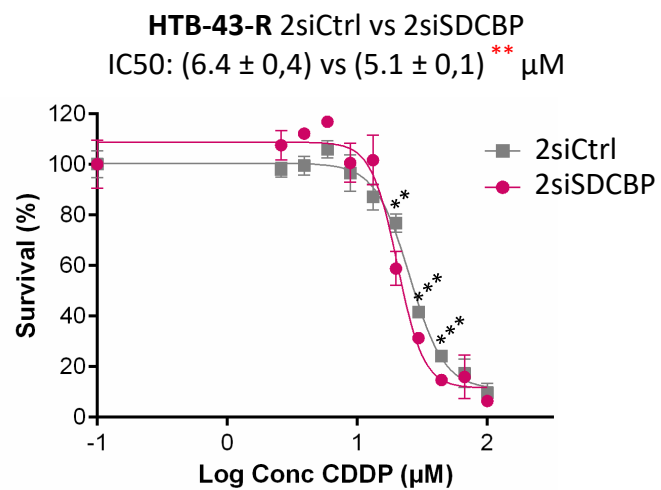

D

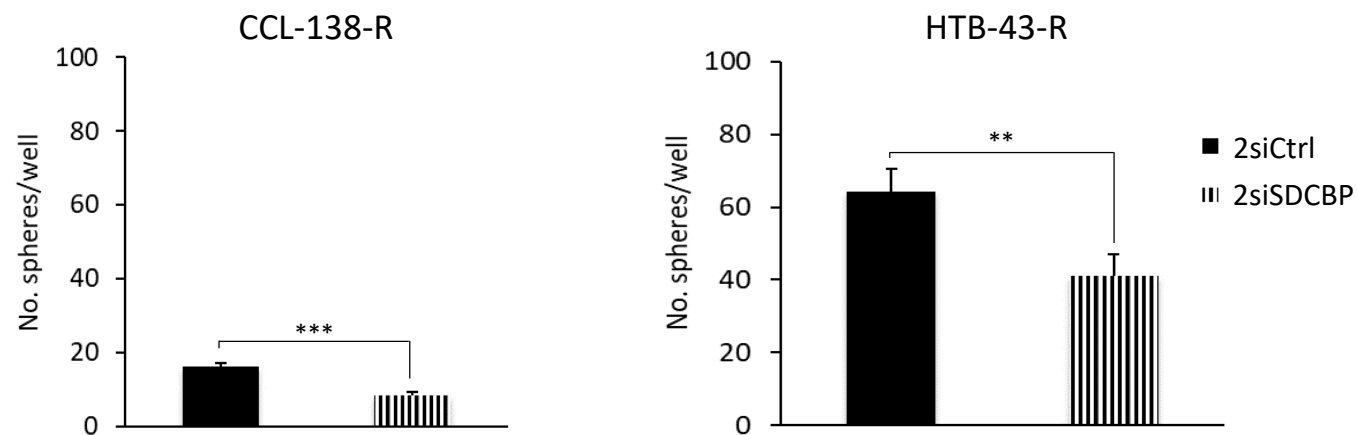

C

| IC50 (μM) |           |           |             |
|-----------|-----------|-----------|-------------|
| Cell line | 2siCtrl   | 2siSDCBP  | % Reduction |
| HTB-43-R  | 6.4 ± 0.4 | 5.1 ± 0.1 | 20.3        |

Figure S5

Supplement: Supplementary file 1 [file cancers-13-04952-s001.zip › Supplementary Figure 5 07-09-21.pdf]

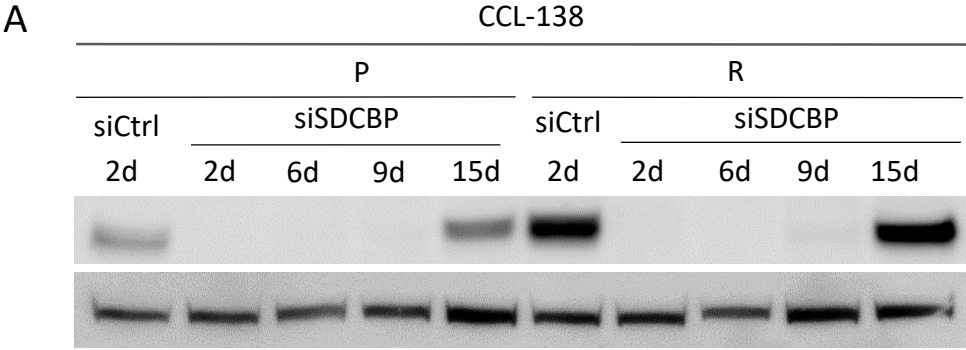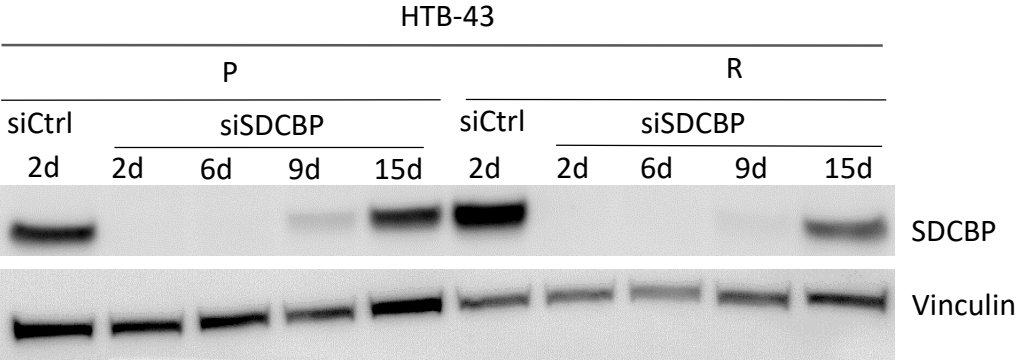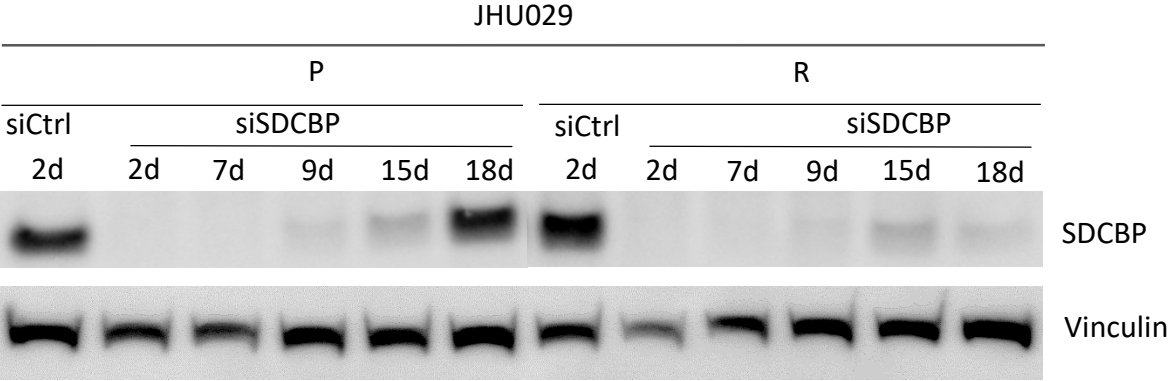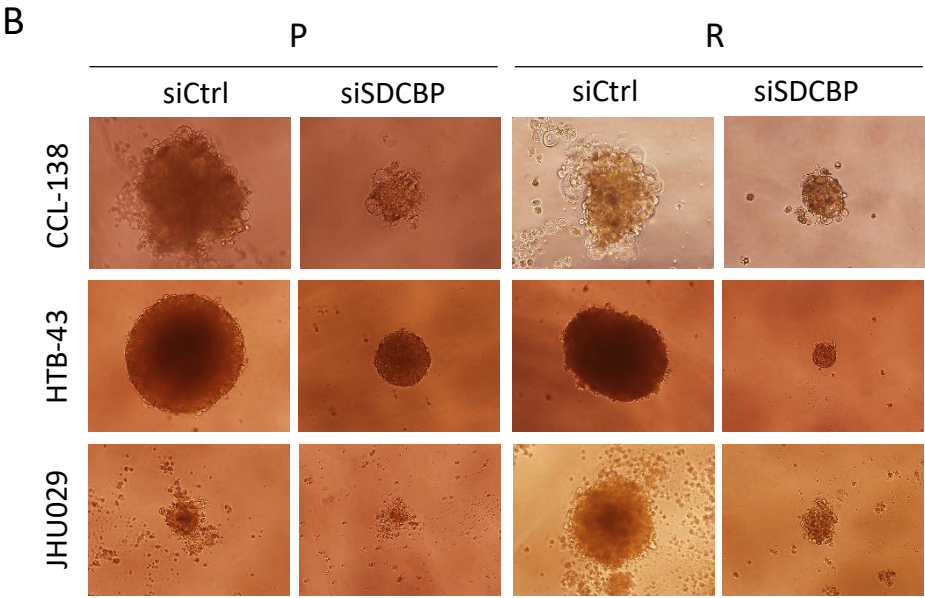

Figure S6

Supplement: Supplementary file 1 [file cancers-13-04952-s001.zip › Supplementary Figure 6 19-07-21.pdf]

# ABCB1

A

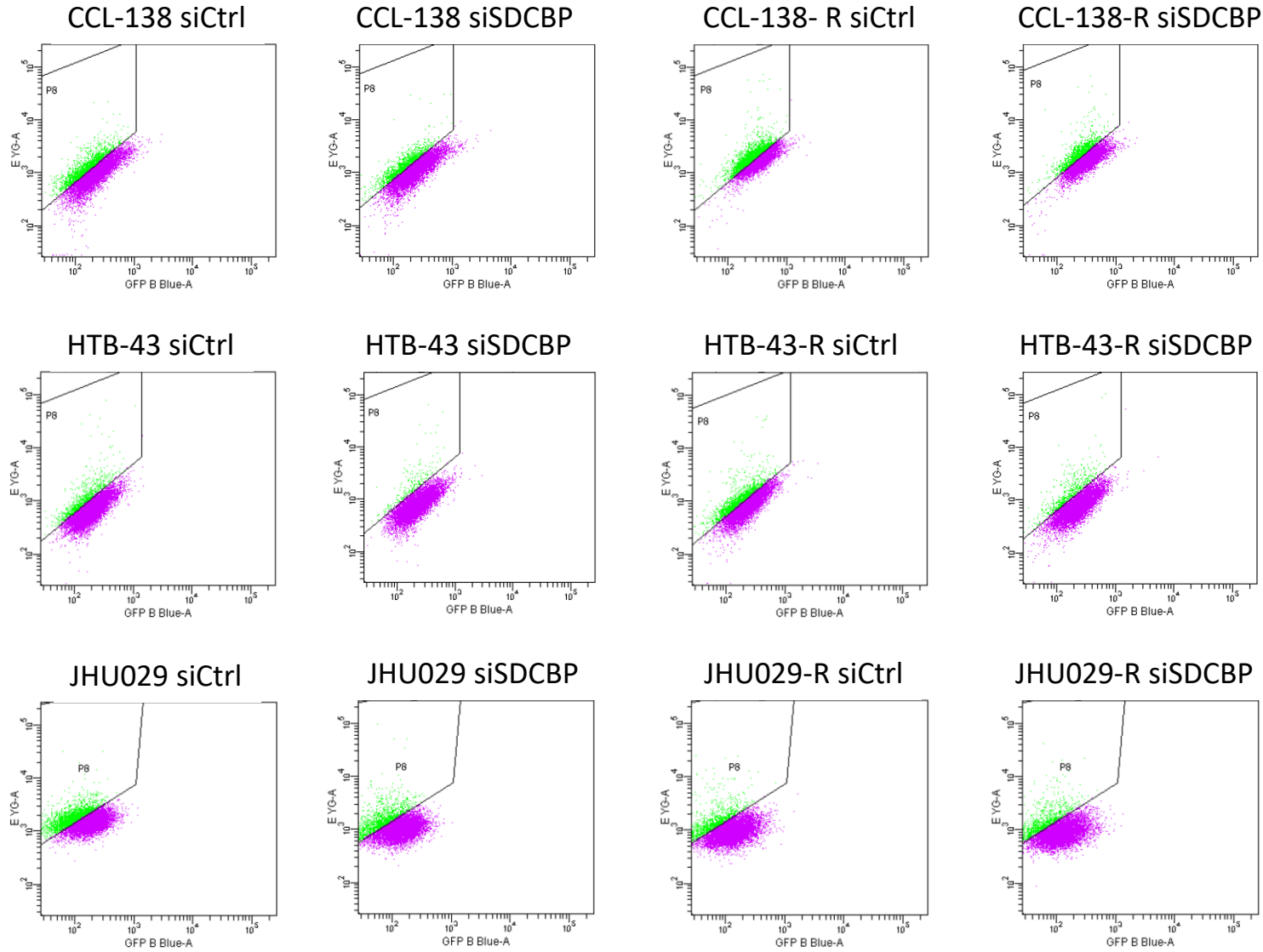

B

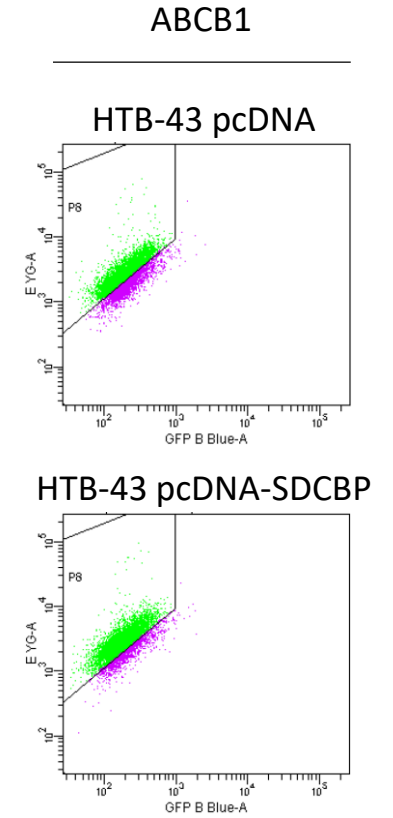

Figure S7

Supplement: Supplementary file 1 [file cancers-13-04952-s001.zip › Supplementary Figure 7 19-07-21.pdf]

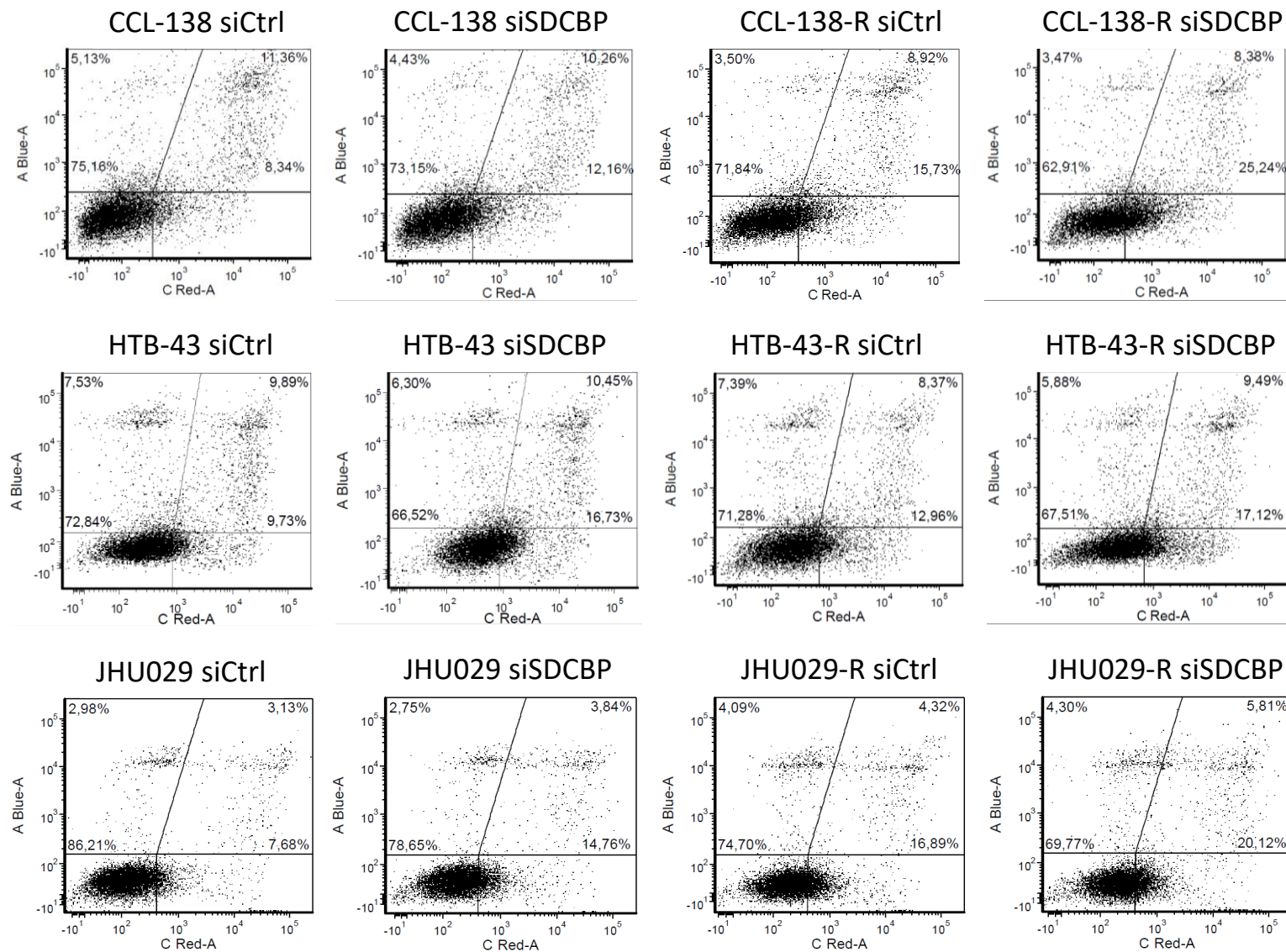

Figure S8

Supplement: Supplementary file 1 [file cancers-13-04952-s001.zip › Supplementary Figure 8 19-07-21.pdf]

A

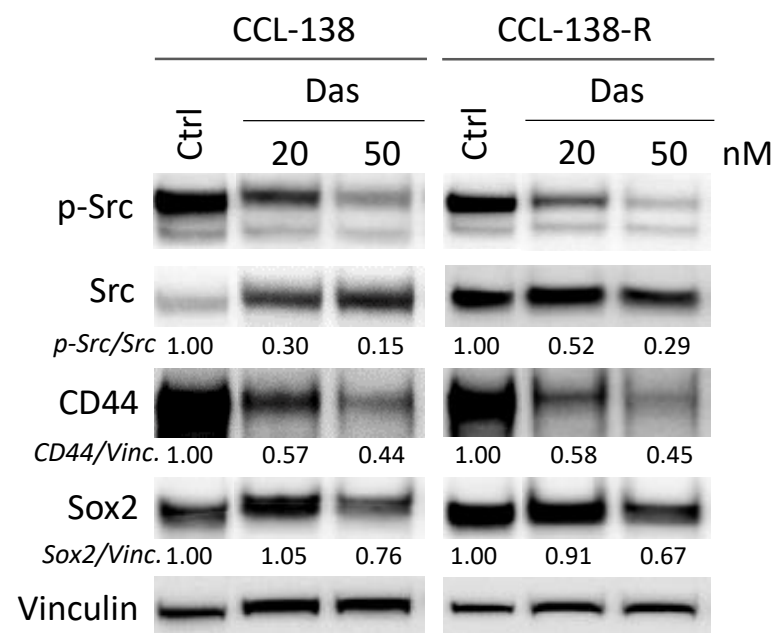

B

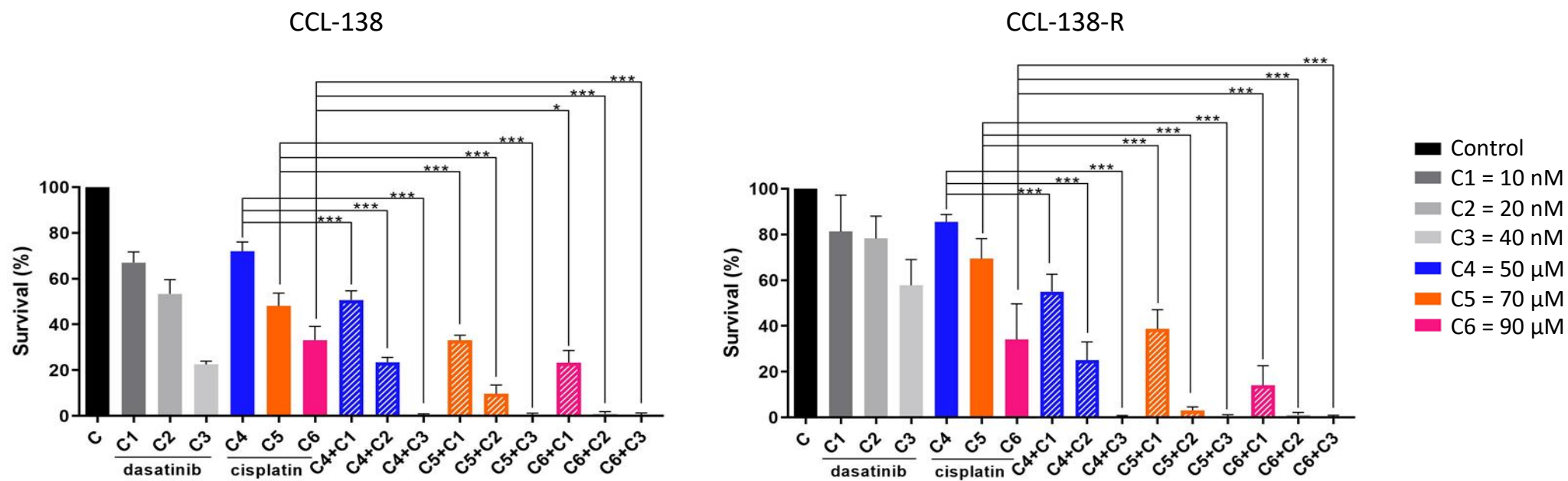

Figure S9

Supplement: Supplementary file 1 [file cancers-13-04952-s001.zip › Supplementary Figure 9 27-08-21.pdf]
